# Qingzhu granule mitigates acute gouty arthritis through HIF-1α-dependent glycolytic metabolic reprogramming of M1 macrophages

**DOI:** 10.3389/fimmu.2026.1817105

**Published:** 2026-08-26

**Authors:** Yulin Qi, Wenwen Li, Qing Wang, Shuiping Zhou, Yuhong Li, Lin Li

**Affiliations:** 1 Key Laboratory of Traditional Chinese Medical Pharmacology, Tianjin University of Traditional Chinese Medicine, Tianjin, China; 2 State Key Laboratory of Chinese Medicine Modernization, Tasly Pharmaceutical Group Co. Ltd, Tianjin, China; 3 Tianjin Key Laboratory of Component-Based Chinese Medicine, Tasly Pharmaceutical Group Co. Ltd, Tianjin, China; 4 Ministry of Education Key Laboratory of Pharmacology of Traditional Chinese Medical Formulae, Tianjin University of Traditional Chinese Medicine, Tianjin, China; 5 State Key Laboratory of Chinese Medicine Modernization, Tianjin University of Traditional Chinese Medicine, Tianjin, China

**Keywords:** acute gouty arthritis (AGA), glycolysis, HIF-1α, M1 macrophage polarization, Qingzhu granule (QZG)

## Abstract

**Background:**

Acute gouty arthritis (AGA) is an acute inflammatory disorder triggered by the deposition of monosodium urate crystals (MSU) in the joints, clinically characterized by severe pain and transient limitation of joint mobility. Without proper intervention, recurrent episodes can lead to irreversible joint deformities, posing a serious threat to the health of patients. Qingzhu granule (QZG), a traditional Chinese medicinal formula, has been clinically applied in the management of AGA, yet its underlying anti-inflammatory mechanisms remain poorly understood.

**Aim of the study:**

This study aims to investigate the therapeutic effects of QZG on MSU-induced AGA and to elucidate its underlying anti-inflammatory mechanisms.

**Methods:**

The AGA mouse model was established via intra-articular MSU injection. Swelling index measurement, histopathological staining and pro-inflammatory factor tests (IL-1β, TNF-α and IL-6) were employed to elucidate the therapeutic effects of QZG on AGA. Bioactive components of QZG were identified using UPLC-Q-TOF-MS analysis, while network pharmacology and bioinformatics approaches were employed to predict its potential targets and pathways. Subsequently, flow cytometry and immunofluorescence were applied to determine the proportion of M1 and M2 macrophages in the ankle joints of AGA mice. MSU-stimulated RAW264.7 cells were used to further verify the direct effect of QZG on M1 macrophage polarization. After detecting the effects of QZG on glycolysis and oxidative phosphorylation in macrophages, the expression of glycolysis-related enzymes was also examined. The potential upstream targets of glycolysis-related genes regulated by QZG were predicted using protein-protein interaction network analysis, random forest algorithm, and ChEA3 database. Finally, HIF-1α overexpression plasmids were utilized to confirm that HIF-1α serves as a key target mediating the regulatory effects of QZG.

**Results:**

QZG significantly alleviated ankle swelling, reduced immune cell infiltration, and decreased pro-inflammatory factor levels (IL-1β, TNF-α and IL-6) in MSU-induced AGA mice. UPLC-Q-TOF-MS analysis identified 30 bioactive constituents in QZG. Network pharmacology and bioinformatics analysis suggested that macrophage polarization may be the key pathway for the anti-inflammatory effects of QZG. QZG effectively reduced the proportion of M1 macrophages in ankle joints of AGA mice, which was further validated in MSU-stimulated RAW264.7 cells. Moreover, glycolysis capacity was decreased after QZG intervention in RAW264.7 cells. Meanwhile, QZG suppressed the mRNA and protein expression of glycolysis-related enzymes *in vivo and in vitro.* Notably, HIF-1α was identified as a key regulatory target of QZG. Upon overexpression of HIF-1α, the inhibitory effects of QZG on macrophage M1 polarization and glycolysis were partially reversed, further confirming the pivotal role of HIF-1α in mediating the pharmacological actions of QZG.

**Conclusion:**

QZG alleviates MSU-induced local inflammation in AGA by suppressing HIF-1α-dependent glycolysis and M1 macrophage polarization.

## 1 Introduction

Acute gouty arthritis (AGA) is an acute inflammatory condition triggered by the deposition of monosodium urate (MSU) crystals in joints, resulting from dysregulated purine metabolism (1). Clinically, it typically presents with joint redness, swelling, pain, and functional impairment (2). A recent report in *Lancet Rheumatology* indicates that the global incidence of gout has continued to rise from 1990 to 2020, with studies projecting that the number of gout patients worldwide will exceed 40 million by 2050 (3). Current management of AGA centers on anti-inflammatory and analgesic strategies, with anti-inflammatory drugs serving as the therapeutic cornerstone (4). However, despite their efficacy in alleviating symptoms, the clinical utility of these agents is constrained by significant limitations. Particular concerns arise for patients with comorbidities, in whom inappropriate drug use may exacerbate pre-existing conditions (5). Consequently, the development of novel anti-AGA agents with enhanced efficacy and broader clinical applicability has attracted considerable research attention in recent years.

Sterile inflammation triggered by MSU crystals is a well-established pathogenic factor in AGA (4). Upon exposure, MSU crystals activate the innate immune system, promoting the release of pro-inflammatory cytokines such as interleukin-1β (IL-1β), thereby initiating an inflammatory response (6). Clinical studies have confirmed that inhibiting the production of these pro-inflammatory cytokines can effectively alleviate symptoms such as redness, swelling, and pain in the ankle joints of AGA patients (7). Within the inflammatory regulatory network underlying AGA, immune cells play a pivotal role, with macrophages being recognized as a central driver of the inflammatory process. As resident immune cells within the joint cavity, macrophages recognize MSU crystals via their surface Toll-like receptors 2 and 4 (TLR2/4) (8). This recognition activates downstream signaling pathways and induces a reprogramming of cellular energy metabolism, driving macrophage polarization toward the pro-inflammatory M1 phenotype, which in turn amplifies the secretion of pro-inflammatory cytokines and promotes the initiation and progression of AGA (9, 10). Previous experimental studies have demonstrated that inhibiting M1 macrophage polarization markedly reduces the levels of inflammatory factors in the ankle joints of AGA mice (11). Therefore, targeting M1 macrophage polarization has emerged as a promising therapeutic strategy for AGA.

Qingzhu granule (QZG) is a traditional Chinese medicine formula composed of Cangzhu (*Atractylodes chinensis (DC.) Koidz.*), Zhishi (*Citrus aurantium L.*), Huzhang (*Polygonum cuspidatum Sieb. et Zucc.*), Huangbai (*Phellodendron chinense Schneid.*), Yimucao (*Leonurus japonicus Houtt.*), Mianbixie (*Dioscorea spongiosa J.Q. Xi, M. Mizuno et W. L. Zhao*), Zaojiaci (*Gleditsia sinensis Lam.*), Qingfengteng (*Sinomenium acutum (Thunb.) Rehd. et Wils.*), and Danshen (*Salvia miltiorrhiza Bge.*). Clinical evidence indicates that QZG effectively alleviates joint swelling and improves restricted mobility in AGA patients (12). Experimentally, multiple active components in QZG have demonstrated anti-AGA properties. Phellodendrine from *Phellodendron chinense* (Huangbai) has been shown to ameliorate gouty arthritis by suppressing the IL-6/STAT3 signaling pathway (13). Polydatin from *Polygonum cuspidatum* (Huzhang) alleviated ankle swelling in MSU-treated mice by reducing levels of nitric oxide and malondialdehyde (14). Furthermore, naringenin from *Citrus aurantium* (Zhishi) attenuated MSU-induced macrophage inflammation via inhibition nuclear factor κB (NF-κB) (15). Despite these promising findings, the comprehensive anti-AGA mechanism of QZG remains incompletely elucidated.

In this study, we constructed an MSU-induced mouse AGA model via intra-articular injection of MSU crystals to evaluate the therapeutic efficacy of QZG. Subsequently, UPLC-Q-TOF-MS was employed to identify the active constituents in QZG, and network pharmacology along with bioinformatics approaches were integrated to systematically predict its potential mechanisms and signaling pathways against AGA. Furthermore, flow cytometry and Seahorse XF analyzer were utilized to delineate the regulatory role of QZG in macrophage M1 polarization and glycolytic metabolism. Based on these findings, protein-protein interaction (PPI) network analysis and random forest algorithm were combined to identify the core targets of QZG in AGA treatment, which were subsequently validated both *in vivo* and *in vitro*. Finally, by constructing hypoxia-inducible factor-1α (HIF-1α)-overexpressing RAW264.7 cells, we further clarified the role of HIF-1α in the mechanism by which QZG targets glycolysis to inhibit M1 macrophage polarization.

## 2 Materials and methods

### 2.1 Chemicals and reagents

QZG was provided by Tasly Pharmaceutical Group Co. Ltd (China). Colchicine was obtained from Guangdong Pidi Pharmaceutical Co., Ltd (China). MSU was purchased from Sigma-Aldrich (USA). Antibodies against hexokinase2 (HK2) (DF6176), pyruvate kinase isozyme type M2 (PKM2) (AF5234), phosphofructokinase (PFKM) (AF7562), and lactate dehydrogenase A (LDHA) (DF6280) were purchased from Affinity Biosciences (Suzhou, China). Antibodies against HIF-1α (36169T) and glucose transporter 1 (GLUT1) (12939T) were purchased from Cell Signaling Technology. Bicinchoninic acid (BCA) protein assay kit and RIPA buffer were obtained from the Beijing Solarbio Science & Technology Co., Ltd. (Beijing, China). Hieff UNICON^®^ universal blue qPCR SYBR green master mix and all-in-one first-strand cDNA synthesis supermix for qPCR kit were purchased from Yeasen Biotechnology (Shanghai, China).

### 2.2 Animals and treatments

All animal procedures were approved by the Animal Ethics Committee of Tianjin University of TCM (Ethics Approval No. TCM-LAEC2025088q1925). Male C57BL/6 mice (6–8 weeks old) were obtained from Beijing Vital River Laboratory Animal Technology Co., Ltd. (Beijing, China) and maintained under a 12 h light/dark cycle with free access to food and water.

Following a one-week acclimatization period, mice were randomly allocated into five groups (n=8 per group): control (CON) group, MSU group, low-dose QZG (QZG-L, 1.6 g/kg/day) group, high-dose QZG (QZG-H, 3.2 g/kg/day) group, and colchicine (1 mg/kg/day) group. The doses of QZG for mice were calculated by body surface area-based human-mouse dose conversion (Nair et al., 2016). All treatment groups received oral administration once a day for 4 days, while mice in the CON and MSU groups received an equal volume of ultra-pure water. On the fourth day, all mice except the CON group received an intra-articular injection of 25 μL MSU (50 mg/mL) into the left ankle joints to induce AGA, while CON mice received an equal volume of normal saline. Degree of ankle swelling was observed for 24 h after injection. At the end of the experiment, mice were anesthetized with tribromoethanol (400 mg/kg). Following deep anesthesia, mice were euthanized by CO_2_ inhalation. Ankle joints were then collected, snap-frozen in liquid nitrogen, and stored at -80 °C for subsequent analysis.

### 2.3 UPLC-MS/MS analysis

UPLC-MS/MS analysis was carried out on an ACQUITY UPLC system equipped with Waters ACQUITY UPLC^®^ HSS T3 (2.1×100 mm, 1.8 μm). The column temperature, sample injection volume, and flow rate were 30 °C, 2 µL, and 0.3 mL/min, respectively. The mobile phase consisted of solvent A (acetonitrile) and solvent B (0.2% formic acid in water). Chemical constituents were separated with the following gradient: 0–2 min, 95%B; 2–30 min, 95%-73%B; 30–34 min, 73%-10%B; 34–36 min, 10%B; 36–37 min, 10-95%B; 37–40 min, 95%B. The electrospray ionization source (ESI) and full-information tandem mass spectrometry (MSE) were used for scanning in positive and negative ion modes, with a scanning range of m/z 50-1200. The capillary voltage was 2.3 kV and cone voltage was 40 V.

### 2.4 Swelling index measurement

The ankle joint circumference of mice was measured prior to MSU injection and at 6, 12, and 24 h post-injection. Ankle swelling index of mice was calculated as follows:

Swelling index (%) =(VAfter MSU injection − VBefore MSU injection)/VBefore MSU injection× 100%.

### 2.5 Enzyme-linked immunosorbent assay

Concentrations of IL-1β, TNF-α and IL-6 in ankle joints of mice were determined by using commercial enzyme-linked immunosorbent assay (ELISA) kits (Suzhou Grace Biotechnology Co., Ltd) according to the manufacturer protocol.

### 2.6 Differential gene acquisition and pathway enrichment analysis

The gout-related gene microarray datasets GSE190138 and GSE160170 were obtained from the GEO database. Differentially expressed genes (DEGs) were screened using R software. Significance cutoffs were determined as follows: (|log_2_FC|>1 and adj P value<0.05). Subsequently, the active ingredients of QZG were submitted to the SwissTargetPrediction platform for target prediction. The predicted targets from QZG ingredients were intersected with the DEGs identified from GSE190138 to construct a target gene dataset for QZG in AGA treatment (QZG-AGA DEGs). DEGs from GSE160170 were used to validate the reliability of the QZG-AGA DEGs. Pathway enrichment analysis of QZG-AGA DEGs was performed using Ingenuity Pathway Analysis (IPA).

### 2.7 Screening of upstream transcription factors

Glycolysis-related genes, including *Glut1, Hk2, Pfkm, Pkm2, Ldha*, and *Pfkfb3* were submitted to the ChEA3 database (https://maayanlab.cloud/chea3/). Potential upstream transcriptional regulators were identified and filtered based on mean rank.

### 2.8 Flow cytometry

To investigate the regulatory effect of QZG on MSU-stimulated macrophage polarization, cells were stained by anti-F4/80, anti-CD86 and anti-CD206. The stained cells were analyzed by flow cytometry (Attune NxT, Thermo Fisher Scientific).

### 2.9 QZG-containing serum preparation

Human clinical equivalent doses were converted to rat doses via body surface area scaling, and the maximum tolerable rat dose was chosen to maximize *in vivo* blood drug levels. Subsequently, healthy rats were randomly divided into two groups: control serum group and QZG-containing serum group (6.4 g/kg). Rats in QZG-containing serum group were administered QZG suspension by gavage at 10 mL/kg (twice/day, for 3 days). Blood samples were collected from the abdominal aorta 2 h after the final administration. The blood was centrifuged at 4 °C to isolate the serum. The serum samples were heat-inactivated at 56 °C for 30 min. After sterilization by filtration through a 0.22 μm membrane, the serum samples were stored at -80 °C for subsequent cell experiments.

### 2.10 Cell culture and treatments

RAW264.7 cells were obtained from ATCC and cultured with Dulbecco’s Modified Eagle Medium (DMEM) supplemented with 10% fetal bovine serum (FBS) at 37 °C in a humidified incubator with 5% CO_2_. Cells were seeded into 6-well plates (1×10^6^cells/well) and divided into four groups: CON, MSU, low-dose QZG (0.6%), and high-dose QZG (1.2%). When cells reached 80-90% confluence, they were treated with drug-containing serum prepared from different concentrations of QZG for 12 h. Subsequently, all groups except for the CON group were stimulated with MSU (200 μg/mL) for 10 h to establish a pro-inflammatory macrophage model. Cells were then collected for protein and RNA extraction.

### 2.11 Cell viability assay

Cell Counting Kit-8 (CCK-8) assay was used to determine the non-cytotoxic concentration of QZG-containing serum. RAW264.7 cells were seeded in 96-well plates (2×10^4^cells/well) and treated for 24 h with various concentrations of the serum (0.3%, 0.6%, 1.2%, 2.5%, 5%, 10%, 15%, and 20%). Cell viability was then assessed according to the manufacturer protocol (Shanghai Beyotime Biotechnology Co., Ltd).

### 2.12 Immunofluorescence staining

To detect the expression of M1 macrophage marker iNOS, RAW264.7 cells were fixed with 4% paraformaldehyde for 30 min, washed with PBS, and permeabilized with 0.1% Triton X-100 on ice for 10 min. Subsequently, cells were blocked with 5% goat serum for 30 min at room temperature and incubated overnight at 4 °C with a primary antibody against iNOS (1:400). After incubation with secondary antibodies for 1 h at room temperature, DAPI was added for nuclear staining. Finally, the samples were scanned using an Operetta high-content screening system.

### 2.13 Seahorse analysis

The oxygen consumption rate (OCR) and extracellular acidification rate (ECAR) were measured using a Seahorse XFe96 Analyzer (Agilent Technologies) to assess oxidative phosphorylation and glycolytic function. RAW264.7 cells were seeded in Seahorse XF96 cell culture microplates (1×10^4^cells/well) and cultured overnight to allow attachment. After treatment with QZG and MSU, cells were incubated in Seahorse assay medium with substrate supplementation for 40 min at 37 °C in a CO_2_-free incubator. For the oxidative phosphorylation test, three baseline OCR measurements were taken, followed by sequential injections of oligomycin (1 µM), FCCP (2 µM), and a combination of rotenone (0.5 µM) and antimycin A (0.5 µM). For the glycolytic stress test, three baseline ECAR measurements were recorded, followed by sequential injections of glucose, oligomycin, and 2-deoxy-D-glucose (2-DG, 50 mM). All data were normalized to cell number using an automated cell imaging system (Falcon S300, Alicelligent Technologies).

### 2.14 Western blotting

Total proteins were extracted from ankle joint tissue using RIPA buffer supplemented with phosphatase inhibitors and PMSF complex. Protein concentration was determined using the BCA Protein Assay Kit (Solarbio Science & Technology Co., China). Protein samples were separated by SDS-PAGE on an 8% gradient gel and transferred onto PVDF membranes. Membranes were blocked with the protein-free blocking solution and incubated overnight at 4 °C with primary antibodies. Subsequently, membranes were washed repeatedly with TBST and incubated with secondary antibody for 90 min. Finally, protein images were acquired using an Amersham Imager 600 system (GE Healthcare Biosciences AB, Japan). Target protein values were calculated after normalization against β-actin.

### 2.15 Quantitative RT-PCR

Total RNA was isolated from ankle joints samples using TRIzol reagent (Yeasen Biotechnology, Shanghai). RNA concentration and purity were assessed using a Dionex DS-11 spectrophotometer (Dionex Corporation, USA). Subsequently, 1 µg of total RNA from each sample was reverse-transcribed into first-strand cDNA using a cDNA Synthesis Supermix kit (TransGen Biotech, Beijing). Quantitative real-time PCR (qPCR) was then performed using the Hieff UNICON^®^ Universal Blue qPCR SYBR Green Master Mix (Yeasen Biotechnology, Shanghai) on a CFX Connect Real-Time PCR Detection System (Bio-Rad, California, USA). Target gene values were calculated after normalization against β-actin.

### 2.16 *Hif1a* gene overexpression

RAW264.7 cells were seeded in 6-well plates. Upon reaching 90% confluence, the culture medium was aspirated, and the cells were gently washed once with DMEM. Subsequently, 2 mL of DMEM was added to each well, and the cells were cultured for 1 h. For HIF-1α overexpression, a transfection complex was prepared as follows: 4 μg of HIF-1α plasmid was diluted in 250 μL of DMEM and incubated at room temperature for 5 min. In a separate tube, 10 μL of StarFect Lip2000 Transfection Reagent was mixed with 250 μL of DMEM. The two solutions were mixed and incubated at room temperature for 20 min. The complex was then added to the pre-seeded cells. After 12 h of incubation, the medium was replaced, and the cells were further cultured for 24 h. In this study, the RAW264.7 cells overexpressing HIF-1α were pretreated with QZG-containing serum prior to MSU stimulation.

### 2.17 Statistical analysis

Data are presented as mean ± standard error of the mean (SEM). The Shapiro-Wilk test was applied to evaluate the normality of all datasets. Normally distributed data were analyzed by one-way analysis of variance (ANOVA) followed by Tukey’s *post-hoc* test for pairwise comparisons, whereas non-normal datasets were analyzed using the non-parametric Kruskal-Wallis test coupled with Dunn’s *post-hoc* test. In addition, two-group comparisons were performed using independent samples t-test for normally distributed data, and the Mann-Whitney U rank-sum test for data that failed normality testing. All statistical analyses were conducted with GraphPad Prism software. *P* ≤ 0.05 was considered statistically significant.

## 3 Results

### 3.1 QZG relieves the MSU-induced inflammation of ankle joints in AGA mice

**Figure 1:**
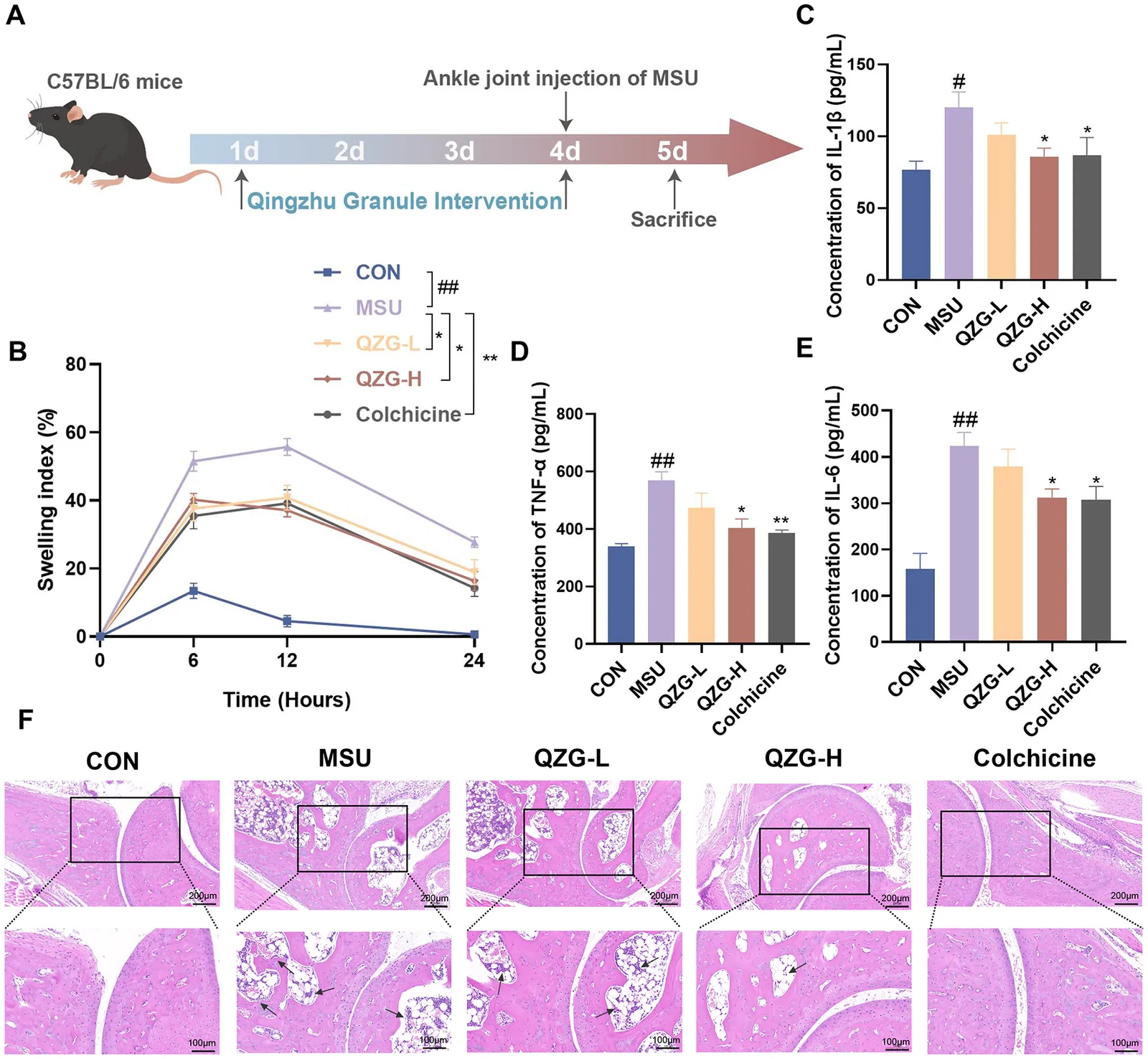
QZG relieves the MSU-induced inflammation of ankle joints in AGA mice. **(A)** The schematic experimental schedule. **(B)** Ankle swelling index (n=6). **(C–E)** Levels of IL-1β, TNF-α and IL-6 in ankle joints were detected by ELISA assay (n=6). **(F)** Representative image of H&E staining in ankle joints. Data are exhibited as mean ± SEM. ^#^*p* < 0.05 and ^##^*p* < 0.01 vs. CON group, *p < 0.05 and **p < 0.01 vs. MSU group.

The anti-AGA effect of QZG was investigated using an MSU-induced AGA mouse model (**Figure 1A**). First, we evaluated the ankle swelling index, a critical indicator reflecting the severity of gout. Compared with control mice, the ankle swelling index was persistently elevated and peaked at 12 h in MSU-treated mice, while this rising trend was significantly suppressed following QZG and colchicine intervention (**Figure 1B**). Consistent with the above findings, MSU-treated mice exhibited significantly higher intra-articular levels of pro-inflammatory cytokines (IL-1β, TNF-α and IL-6) than control mice. QZG treatment effectively suppressed the upregulation of these cytokines in a dose-dependent manner, with colchicine showing a similar inhibitory effect (**Figures 1C–E**). Moreover, histopathological analysis revealed that MSU stimulation disrupted the ankle joint structure and exacerbated immune cell infiltration, both of which were markedly alleviated by QZG and colchicine treatment (**Figure 1F**). Collectively, these data suggest that QZG effectively relieves the MSU-induced inflammation in the ankle joints of AGA mice.

### 3.2 Identification of QZG components

**Figure 2:**
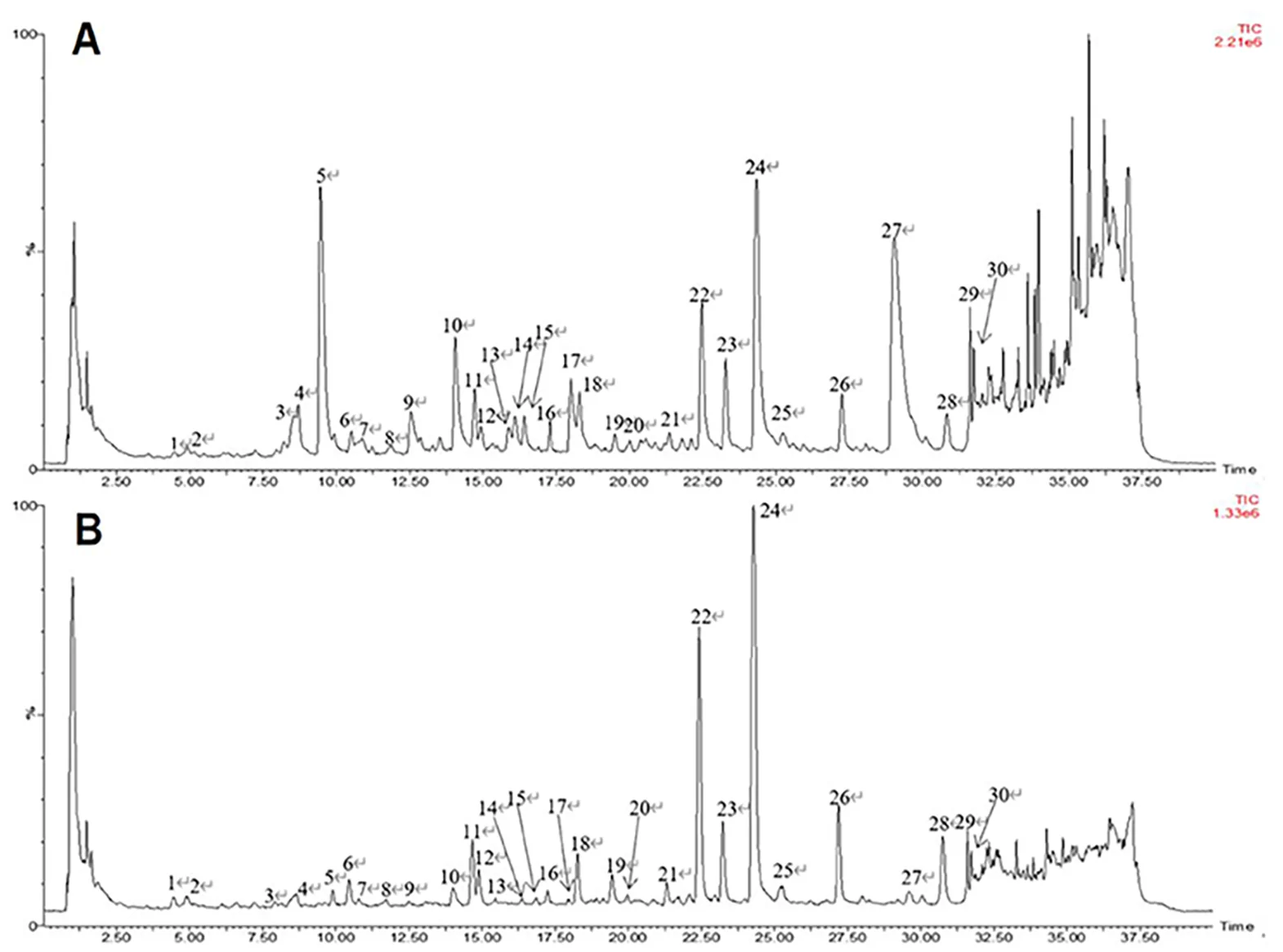
Identification of components of QZG. **(A)** TIC of QZG in positive ion mode. **(B)** TIC of QZG in negative mode.

To characterize functional compounds in QZG, we performed UPLC-Q-TOF-MS chemical fingerprint profiling on QZG. Positive and negative ion modes of UPLC-Q-TOF-MS were used to generate the total ion chromatograms (TICs) of QZG (**Figures 2A, B**). A total of 30 compounds, including but not limited to the phytochemical classes: alkaloids, glucosides, saponins, flavonoids, coumarins and organic acids, were tentatively identified in QZG based on their retention times and mass fragmentation (**Supplementary Table 1**). Among them, 21 components, including higenamine glucoside, sinomenine and phellodendrine, were detected in the positive ion mode, while 9 components, such as dioscorosides B2 and salvianolic acid B were detected in the negative ion mode.

### 3.3 Macrophage polarization may be identified as a potential mechanism for QZG in treating AGA

**Figure 3:**
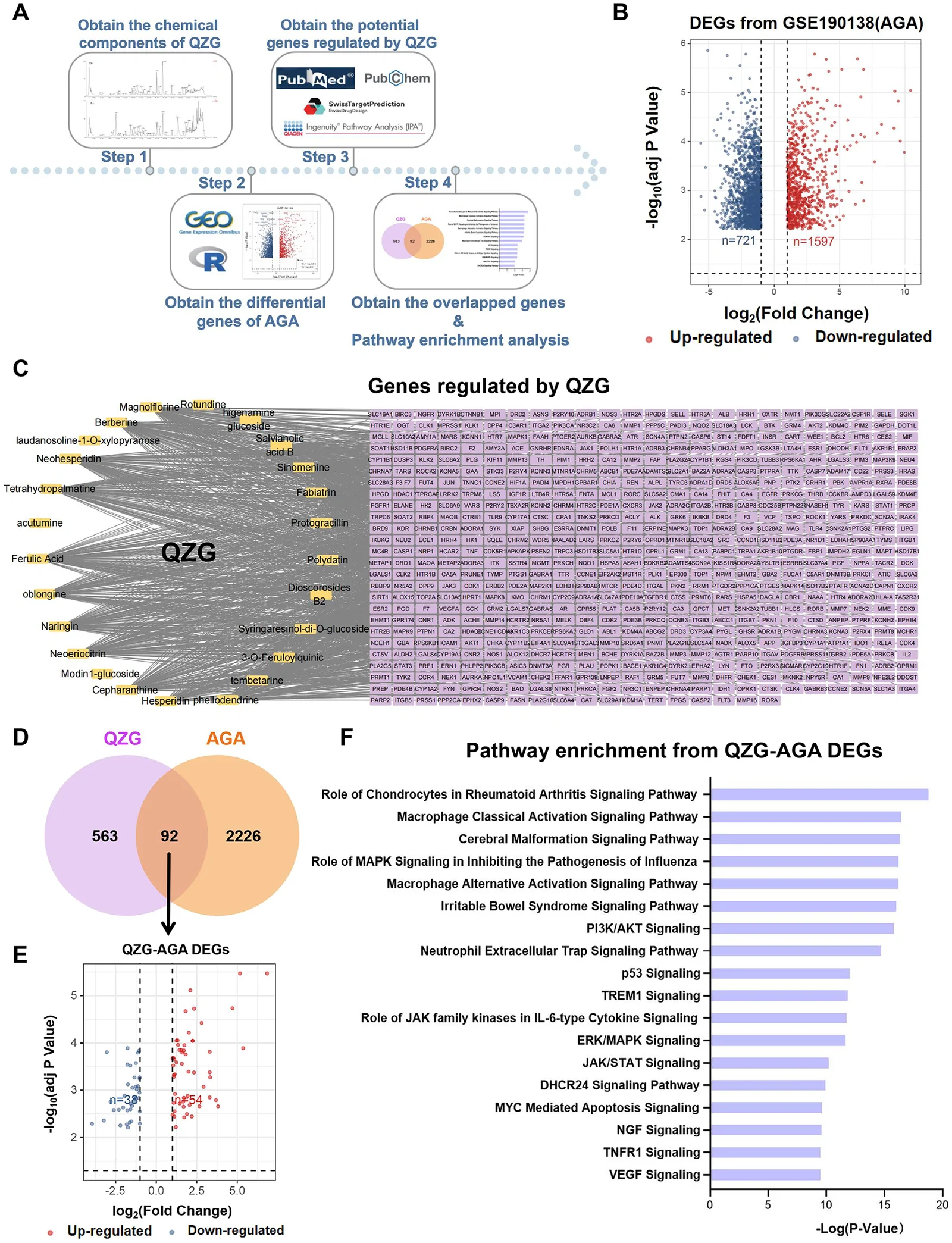
Macrophage polarization may be identified as a potential mechanism for QZG in treating AGA. **(A)** The schematic experimental schedule. **(B)** Volcano plots of DEGs in GSE190138 (|log2FC|>1, adj P value<0.05). **(C)** Component-target network of QZG. **(D)** Venn diagram illustrating the overlapped gene between the DEGs in GSE190138 and QZG-regulated genes. **(E)** Volcano plots of QZG-AGA DEGs (|log2FC|>1, adj P value<0.05). **(F)** Pathway enrichment analysis of the QZG-AGA DEGs from IPA system.

To predict the potential targets and molecular mechanisms by which QZG treats AGA, we carried out network pharmacology and bioinformatic analyses (**Figure 3A**). Differential expression analysis was performed on the acute gout-associated dataset GSE190138, identifying 2318 DEGs: 1597 upregulated and 721 downregulated genes (**Figure 3B**). In addition, a total of 655 QZG-regulated genes were identified by target prediction of 25 active components in QZG after excluding isomers and unknown components (**Figure 3C**). Next, a Venn diagram analysis was conducted between the QZG-regulated genes and the DEGs from GSE190138 to obtain a total of 92 potential genes for QZG against AGA (QZG-AGA DEGs), comprising 54 upregulated genes and 38 downregulated genes (**Figures 3D, E**). Then KEGG pathway enrichment analysis was used to predict the functional attribution of QZG regulation. The top 18 most significant KEGG pathways were selected for scatter plot (**Figure 3F**). Among them, the top 5 ones were Role of Chondrocytes in Rheumatoid Arthritis Signaling Pathway, Macrophage Classical Activation Signaling Pathway (M1 Macrophage polarization), Cerebral Malformation Signaling Pathway, Role of MAPK Signaling in inhibiting the Pathogenesis of Influenza, and Macrophage Alternative Activation Signaling Pathway (M2 Macrophage polarization), which were closely related to inflammation and macrophage activation. Therefore, we assumed that macrophage polarization might be a potential mechanism for QZG in treating AGA.

### 3.4 QZG inhibits M1 macrophage polarization in the ankle joints of AGA mice

**Figure 4:**
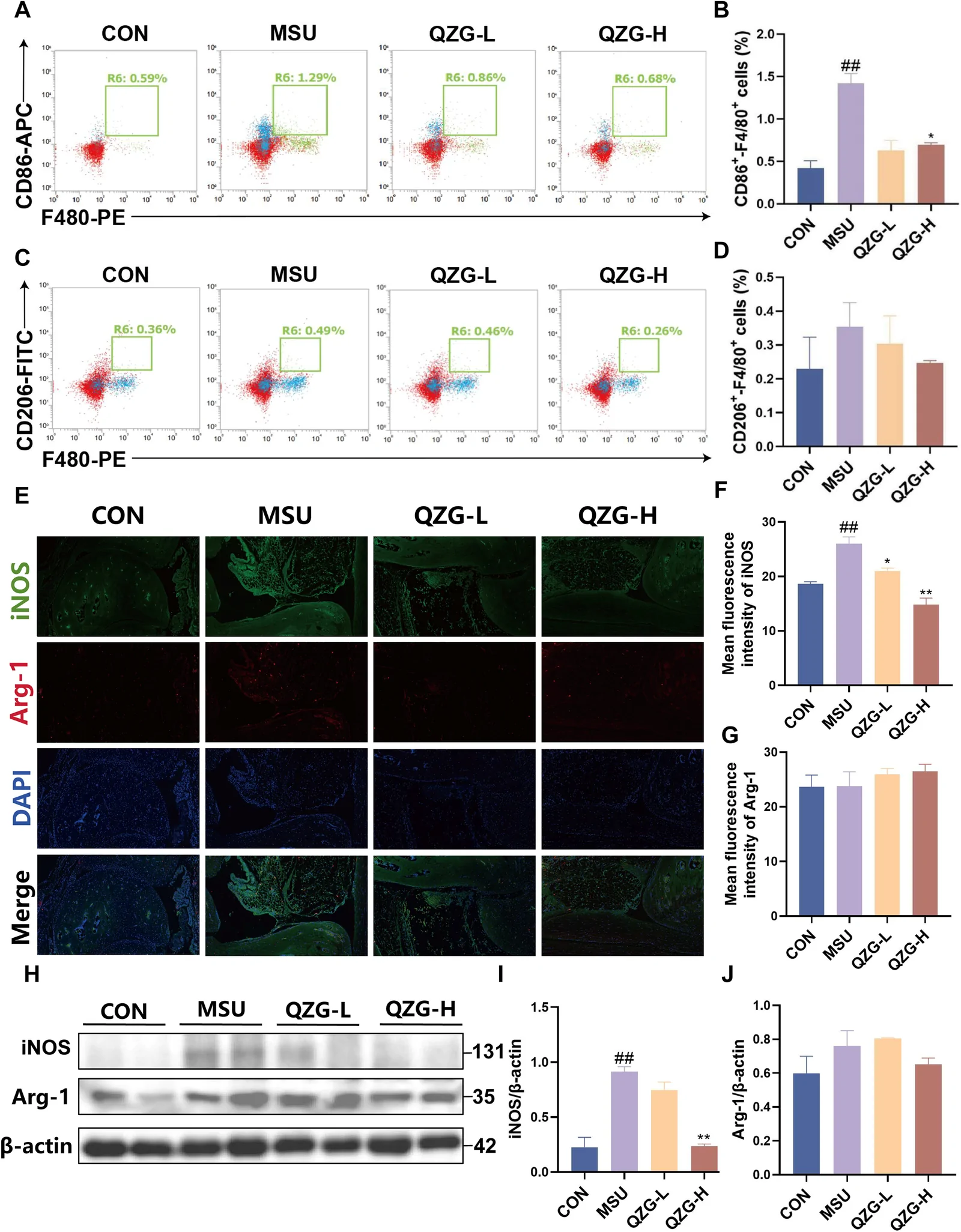
QZG inhibits M1 macrophage polarization in the ankle joints of AGA mice. **(A)** Representative FACS plots of M1 macrophages (CD86^+^-F4/80^+^) in ankle joints. **(B)** Summarized% of M1 macrophages (CD86^+^-F4/80^+^) within gated cell populations (n=3). **(C)** Representative FACS plots of M2 macrophages (CD206^+^-F4/80^+^) in ankle joints. **(D)** Summarized% of M2 macrophages (CD206^+^-F4/80^+^) within gated cell populations (n=3). **(E)** IF staining of iNOS (Green) and Arg-1 (Red) in ankle joints. **(F, G)** Mean fluorescence intensity of iNOS and Arg-1 in ankle joints (n=3). **(H–J)** Protein expression of iNOS and Arg-1 in ankle joints (n=3). Data are exhibited as mean ± SEM. ^#^*p* < 0.05 and ^##^*p* < 0.01 vs. CON group, *p < 0.05 and **p < 0.01 vs. MSU group.

Based on the prediction of pathway enrichment, we determined the levels of macrophage polarization including M1 and M2 in the ankle joints of AGA mice. Flow cytometry analysis indicated that compared with control mice, the percentage of CD86^+^-F4/80^+^ positive cells (M1 macrophages) was elevated in the MSU-treated mice. QZG intervention significantly decreased the percentage of M1 macrophages (**Figures 4A, B**). Meanwhile, no significant difference was noted in the percentage of CD206^+^-F4/80^+^ positive cells (M2 macrophages) for MSU-treated mice with or without QZG administration (**Figures 4C, D**). Similarly, QZG significantly suppressed the MSU-induced upregulation of iNOS (an M1 marker), while it did not affect the downregulation of Arg-1 (an M2 marker) caused by MSU (**Figures 4E–J**). Taken together, these results demonstrate that QZG specifically suppresses M1 macrophage polarization while sparing the M2 phenotype.

### 3.5 QZG reduces the expression of glycolysis-related enzymes in the ankle joints of AGA mice

**Figure 5:**
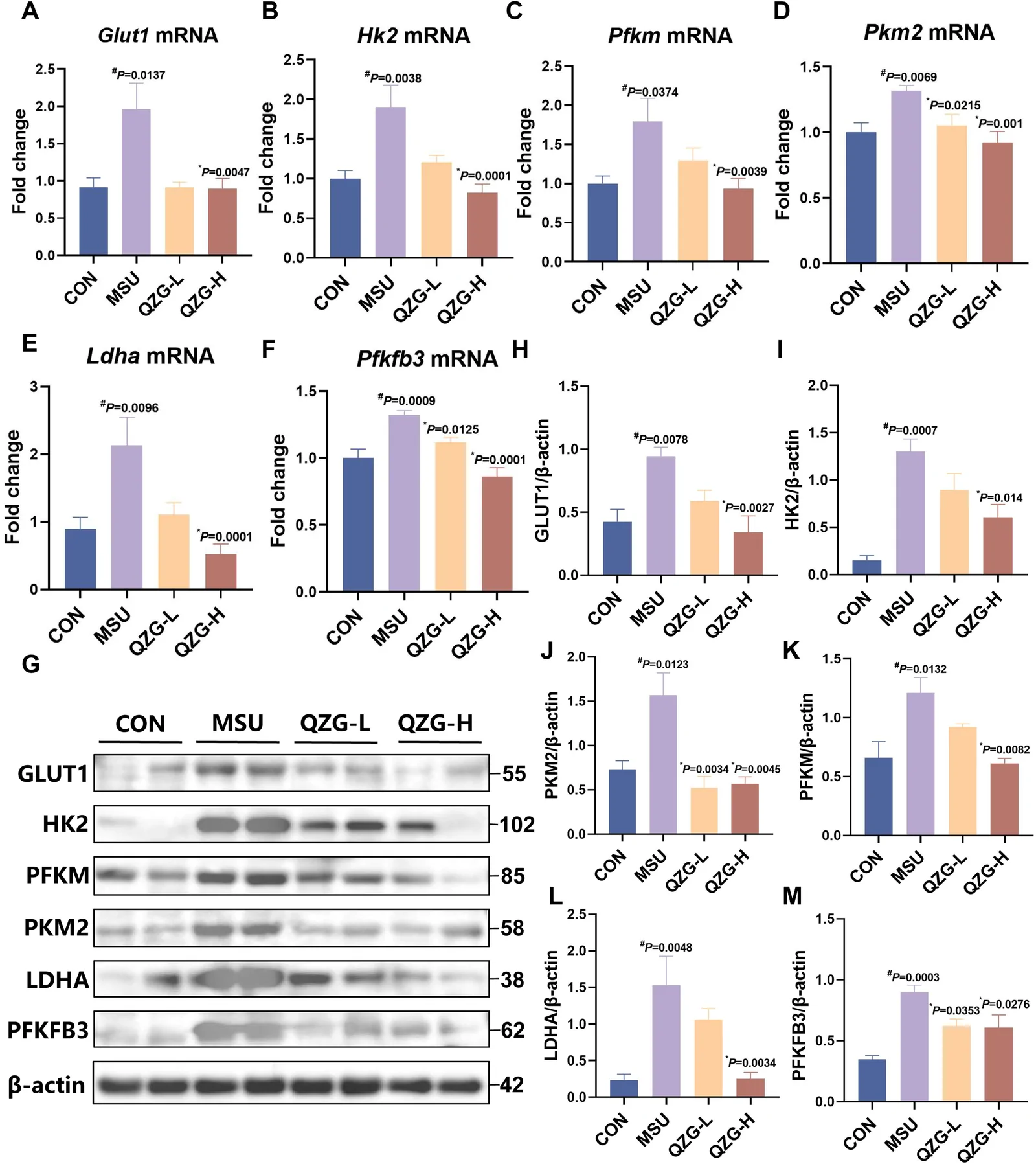
QZG reduces the expression of glycolysis-related enzymes in the ankle joints of AGA mice. **(A–F)** mRNA levels of glycolysis-related enzymes in ankle joints (n=6). **(G–M)** Protein levels of glycolysis-related enzymes in ankle joints (n=3). Data are exhibited as mean ± SEM. ^#^*p* vs. CON group, *p vs. MSU group.

Glycolysis plays a pivotal role in regulating M1 macrophage polarization (16), and the expression level of glycolysis-related enzymes serves as a key determinant of this process (17). Herein, we assessed the effects of QZG on the expression of glycolysis-related enzymes in the ankle joints of MSU-treated mice. As depicted in **Figures 5A–F**, the mRNA levels of *Glut1*, *Hk2*, *Pfkm*, *Pkm2*, *Ldha* and *Pfkfb3* were lower in QZG-treated mice than MSU-treated ones. A consistent suppressive trend was also observed at the protein levels accordingly (**Figures 5G–M**).

### 3.6 QZG inhibits M1 macrophage polarization in MSU-stimulated RAW264.7 cells

Given that MSU serves as a core pathogenic trigger of AGA, we constructed an *in vitro* macrophage inflammatory model using MSU-stimulated RAW264.7 cells to further explore how QZG regulates M1 polarization. We first evaluated the effect of QZG-containing serum on the viability of RAW264.7 cells. The results demonstrated that low concentrations of QZG-containing serum maintained cell viability, and the 1.2% serum group exhibited optimal cell viability. As the concentration of serum continued to rise, cell viability decreased progressively (**Supplementary Figure 1**). On the basis of these cytotoxicity data, we finally selected two non-cytotoxic concentrations (0.6% and 1.2%) for subsequent *in vitro* functional experiments.

**Figure 6:**
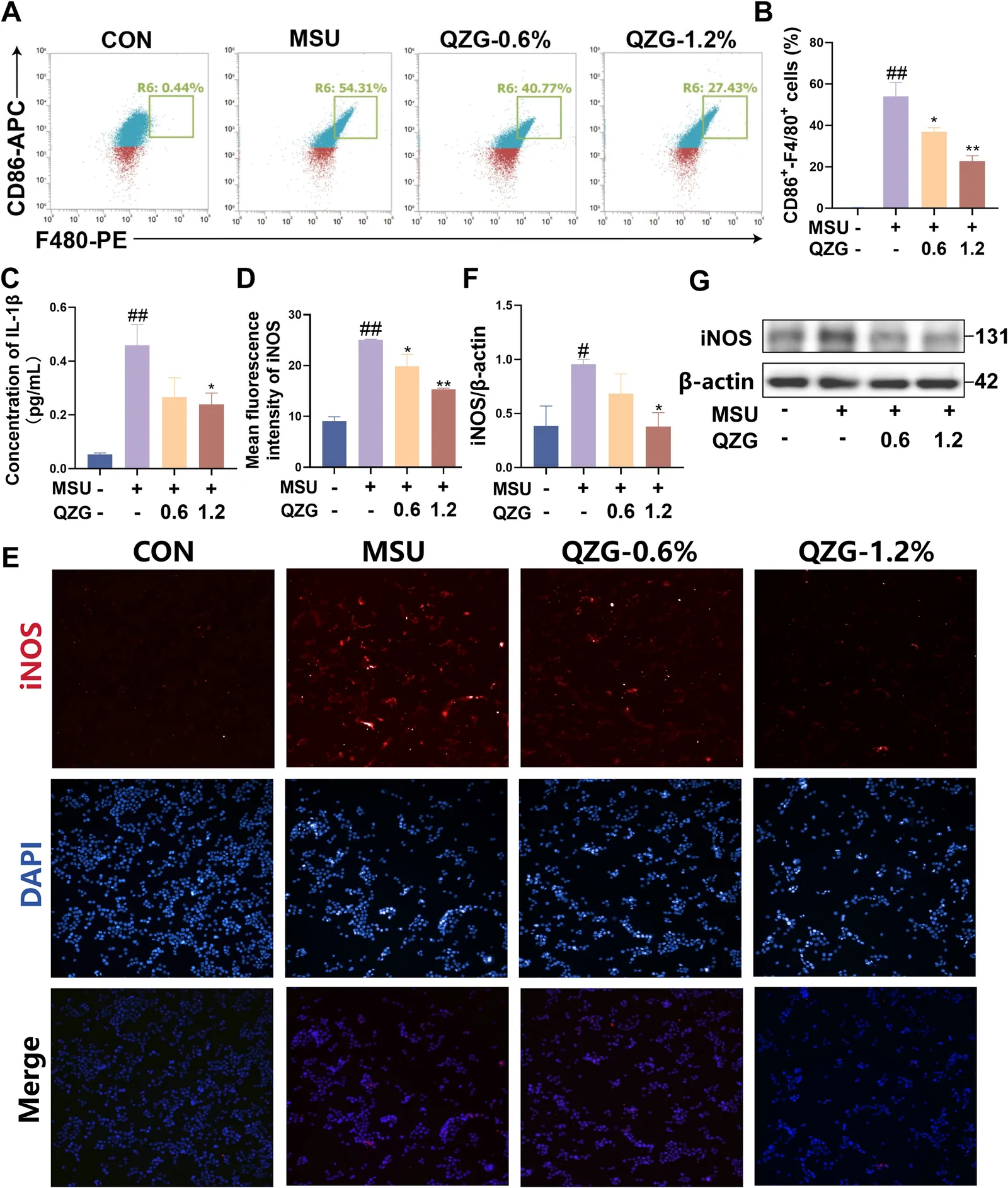
QZG inhibits M1 macrophage polarization in MSU-stimulated RAW264.7 cells. **(A)** Representative FACS plots of M1 macrophages (CD86^+^-F4/80^+^) in RAW264.7 cells (n=3). **(B)** Summarized% of M1 macrophages (CD86^+^-F4/80^+^) within gated cell populations (n=3). **(C)** Levels of IL-1β in RAW264.7 cell culture supernatants were detected by ELISA assay (n=6). **(D)** Mean fluorescence intensity of iNOS (n=3). **(E)** Representative images of IF staining of iNOS (Red). **(F–G)** Western blot analysis of iNOS protein expression in RAW264.7 cells (n=3). Data are exhibited as mean ± SEM. ^#^*p* < 0.05 and ^##^*p* < 0.01 vs. CON group, *p < 0.05 and **p < 0.01 vs. MSU group.

We used flow cytometry to validate the effect of QZG on M1 polarization. Results showed that a lower proportion of CD86^+-^F4/80^+^ positive cells (M1 macrophages) was observed in MSU-stimulated RAW264.7 cells after QZG treatment (**Figures 6A, B**). It is well known that the key pathological function of M1 macrophages lies in the secretion of a series of pro-inflammatory cytokines, including IL-1β, which is a central driver of inflammation in AGA. Accordingly, we measured IL-1β levels in the MSU-induced RAW264.7 cell model. Consistent with our *in vivo* findings, MSU stimulation markedly elevated the IL-1β levels in the cell culture supernatant, and QZG treatment effectively suppressed the upregulation of this cytokine in a dose-dependent manner (**Figure 6C**). Subsequently, we detected the expression of the M1 macrophage marker iNOS. Western blotting and immunofluorescence results showed that iNOS protein abundance was markedly elevated upon MSU stimulation in RAW264.7 cells, while QZG inhibited this upregulation in a dose-dependent manner (**Figures 6D–G**). Collectively, these findings indicate that QZG inhibits M1 macrophage polarization in MSU-stimulated RAW264.7 cells, consistent with the observations *in vivo*.

### 3.7 QZG inhibits macrophage glycolysis in MSU-stimulated RAW264.7 cells

**Figure 7:**
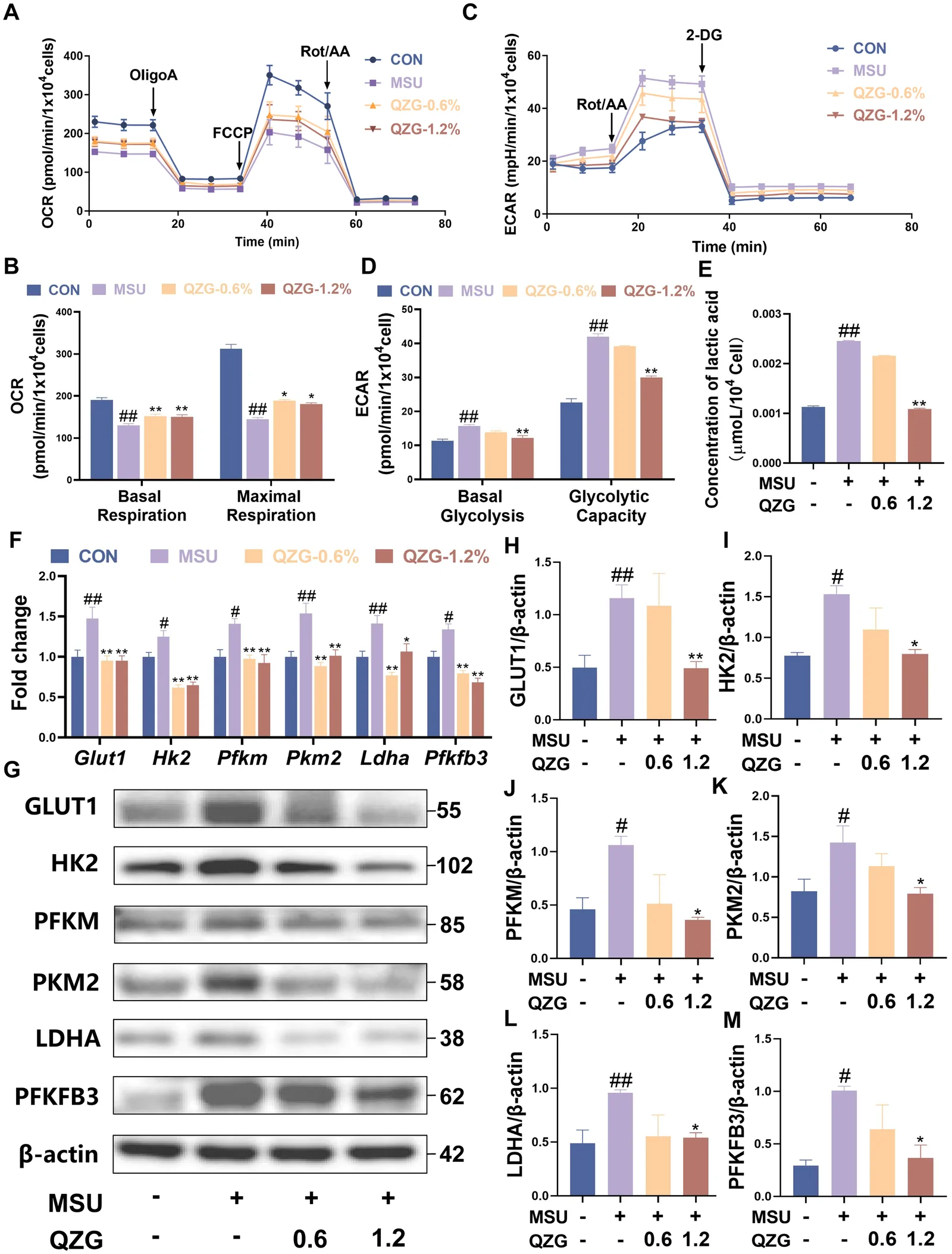
QZG inhibits macrophage glycolysis in MSU-stimulated RAW264.7 cells. **(A)** OCR profile, **(B)** basal respiration, and maximal respiration of RAW264.7 cells (n=6). **(C)** ECAR profile, **(D)** basal glycolysis, and glycolytic capacity of RAW264.7 cells (n=6). **(E)** Levels of lactate of RAW264.7 cells were detected by ELISA assay (n=6). **(F)** mRNA levels of glycolysis-related enzymes in RAW264.7 cells (n=6). **(G–M)** Western blot analysis of glycolysis-related protein expression in RAW264.7 cells (n=3). Data are exhibited as mean ± SEM. ^#^*p* < 0.05 and ^##^*p* < 0.01 vs. CON group, *p < 0.05 and **p < 0.01 vs. MSU group.

Macrophage energy metabolism relies on two distinct pathways: mitochondrial respiration and glycolysis. Evidence indicates that regulating the balance between glycolysis and mitochondrial respiration can effectively prevent the polarization of macrophages to the M1 phenotype. To evaluate the regulatory effect of QZG on energy metabolism of macrophages, we systematically assessed OCR (an indicator of mitochondrial respiration) and ECAR (an indicator of glycolysis). Seahorse analysis revealed that MSU stimulation substantially impaired mitochondrial respiration in RAW264.7 cells, manifesting as marked reductions in both basal and maximal respiration. QZG treatment effectively restored mitochondrial respiratory capacity (**Figures 7A, B**). Conversely, MSU triggered pronounced glycolytic activation, with significant elevations in basal glycolysis and glycolytic capacity. This hyperglycolytic state was robustly suppressed by QZG treatment in a dose-dependent manner (**Figures 7C, D**). Meanwhile, we assessed the levels of lactate (the end product of glycolysis) and key glycolysis-related enzymes. Lower levels of lactate were observed in MSU-stimulated RAW264.7 cells following QZG intervention (**Figure 7E**). Moreover, QZG also remarkably reversed the upregulated mRNA levels of *Glut1*, *Hk2*, *Pfkm*, *Pkm2*, *Ldha* and *Pfkfb3* by MSU stimulation (**Figure 7F**). As expected, a similar trend of protein expression of these glycolysis-related enzymes was observed in QZG-treated RAW264.7 cells (**Figures 7G–M**).

### 3.8 HIF-1α may be the key target of QZG in suppressing glycolysis

**Figure 8:**
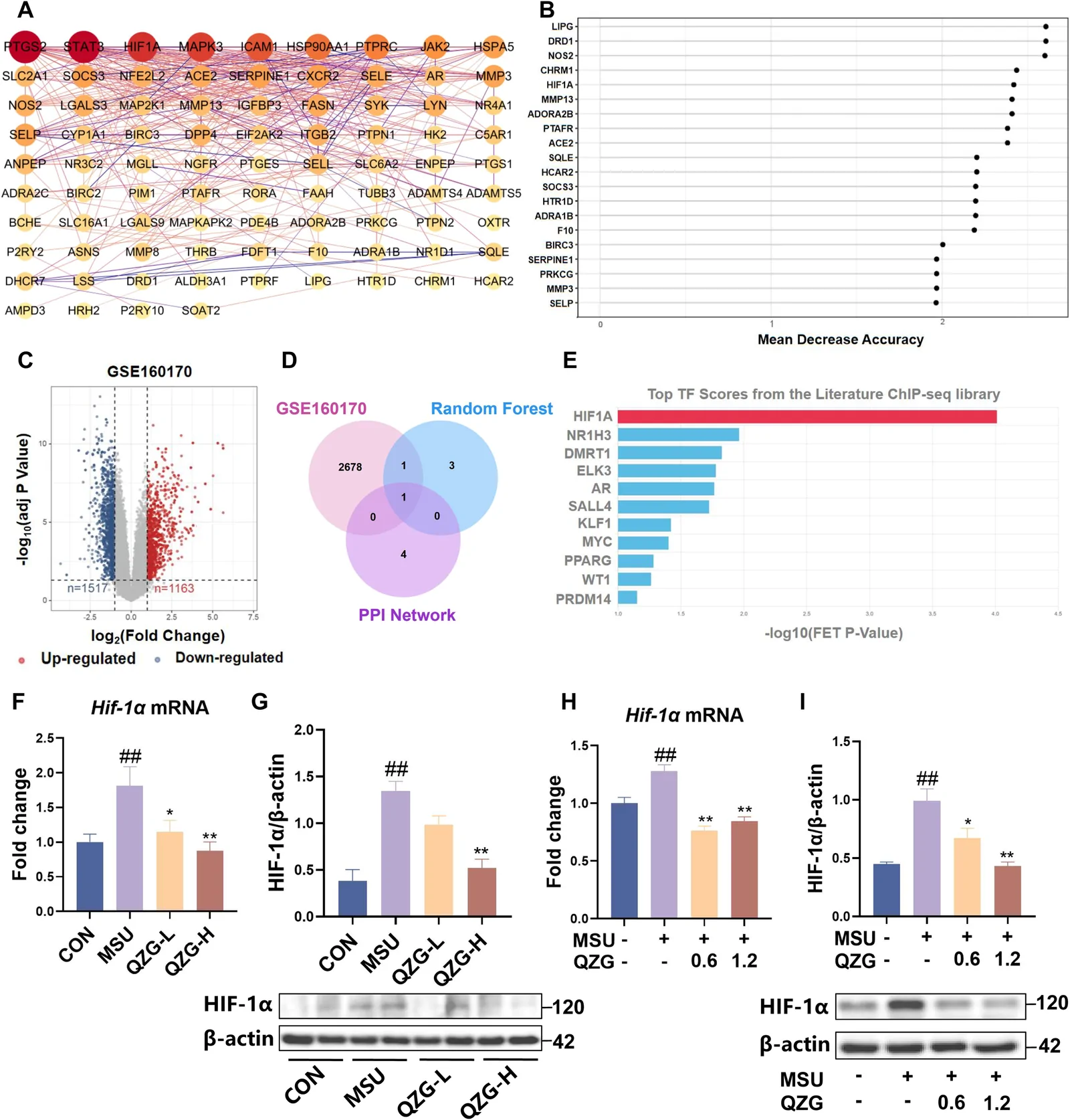
HIF-1α may be the key target of QZG in suppressing glycolysis. **(A)** PPI network analysis to QZG-AGA DEGs. **(B)** Random forest analysis to QZG-AGA DEGs. **(C)** Volcano plots of DEGs in GSE160170. **(D)** Venn diagram illustrating the overlapped genes among QZG-AGA DEGs in PPI network and random forest analysis, and DEGs in GSE160170. **(E)** Prediction of upstream transcription factors for glycolysis-related enzymes using the ChEA3 database. **(F)** *Hif1a* mRNA expression in ankle joints (n=6). **(G)** HIF-1α protein expression in ankle joints (n=3). **(H)** *Hif1a* mRNA expression in RAW264.7 cells (n=6). **(I)** HIF-1α protein expression in RAW264.7 cells (n=3). Data are exhibited as mean ± SEM. ^#^*p* < 0.05 and ^##^*p* < 0.01 vs. CON group, *p < 0.05 and **p < 0.01 vs. MSU group.

To investigate upstream targets of QZG inhibiting the transcription of glycolysis-related enzyme genes, we conducted an integrated bioinformatics analysis. Firstly, protein-protein interaction (PPI) network analysis was performed to screen reliable key genes from QZG-AGA DEGs. According to the degree value, the top five ranked genes were screened, including *Ptgs2, Stat3, Hif1a, Mapk3, and Icam1* (**Figure 8A**). In parallel, a random forest algorithm was applied to screen for critical genes from QZG-AGA DEGs. Interestingly, it identified another set of key genes: *Lipg*, *Drd1*, *Nos2*, *Chrm1*, and *Hif1a* based on mean decrease accuracy (MDA) values (**Figure 8B**).

To validate the candidate genes identified by both PPI and random forest analyses, we analyzed an independent gout microarray dataset (GSE160170). The results showed a total of 2680 DEGs between gout patients and healthy controls, consisting of 1163 up-regulated and 1517 down-regulated genes (**Figure 8C**). Subsequently, Venn overlap analysis was conducted between DEGs from the GSE160170 dataset and the top five hub genes derived from PPI network and random forest analyses. This analysis pinpointed HIF-1α as the sole core regulatory factor (**Figure 8D**). To further confirm the regulatory effect of HIF-1α on the transcription of glycolysis-related enzymes, we analyzed the ChEA3 database and found that HIF-1α was predicted as the most significant upstream regulator of glycolysis-related enzymes (**Figure 8E**).

To validate our bioinformatic prediction, we quantified HIF-1α mRNA and protein levels in ankle joints of MSU-induced AGA mice and MSU-stimulated RAW264.7 cells. QZG treatment markedly reversed MSU-induced upregulation of HIF-1α at both mRNA and protein levels in the ankle joints of AGA mice (**Figures 8F, G**). Consistent with the *in vivo* findings, QZG exerted a comparable suppressive effect on HIF-1α expression in MSU-stimulated RAW264.7 cells (**Figures 8H, I**).

### 3.9 QZG reduces M1 macrophage polarization by inhibiting HIF-1α-mediated glycolysis

**Figure 9:**
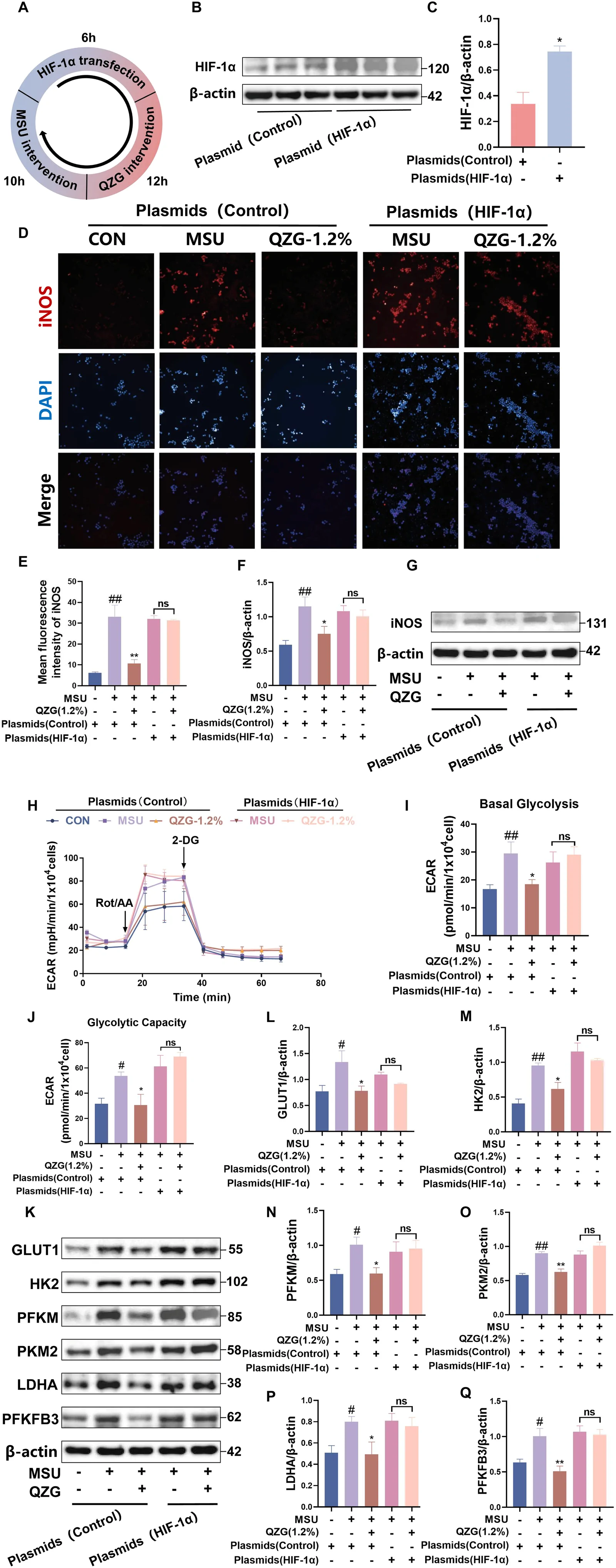
QZG reduces the M1 macrophages polarization by inhibiting HIF-1α mediated glycolysis. **(A)** The schematic experimental schedule. **(B–C)** HIF-1α protein expression in RAW264.7 cells transfected with control plasmids or HIF-1α-overexpressing plasmid (n=3). **(D–E)** Representative images of IF staining of iNOS (Red) and the mean fluorescence intensity of iNOS in the RAW264.7 cells (n=3). **(F–G)** Western blot analysis of iNOS protein expression in the RAW264.7 cells (n=3). **(H)** ECAR profile, **(I)** basal glycolysis, and **(J)** glycolytic capacity of the RAW264.7 cells (n=6). **(K–Q)** Western blot analysis of glycolysis-related protein expression in the RAW264.7 cells (n=3). Data are exhibited as mean ± SEM. ^#^*p* < 0.05 and ^##^*p* < 0.01 vs. plasmid (Control) group, *p < 0.05 and **p < 0.01 vs. plasmid (Control)+MSU group.

To elucidate the role of HIF-1α in QZG-regulated M1 polarization and glycolysis, RAW264.7 cells were transfected with a HIF-1α-overexpressing plasmid. Transfection efficiency was confirmed by Western blotting (**Figures 9A–C**). Under MSU stimulation, the inhibitory effect of QZG on iNOS protein expression was partially reversed upon HIF-1α overexpression (**Figures 9D–G**). Meanwhile, HIF-1α overexpression counteracted the QZG-induced downregulation of glycolysis, as evidenced by enhanced ECAR (elevated basal glycolysis and glycolytic capacity) (**Figures 9H–J**) and glycolysis-related enzymes expression (GLUT1, HK2, PFKM, PKM2, LDHA, and PFKFB3) (**Figures 9K–Q**). Collectively, these findings demonstrate that QZG attenuates M1 macrophage polarization by suppressing HIF-1α-mediated glycolysis.

## 4 Discussion

Despite the well-elucidated pathogenesis of AGA, its global prevalence continues to rise, highlighting the limitations of current clinical therapeutic strategies (1, 18). Substantial evidence confirms that inflammatory response serves as the core pathological driver in the onset and progression of AGA (6). The 2020 American College of Rheumatology (ACR) guidelines emphasized anti-inflammatory therapy as a cornerstone of AGA clinical management. However, first-line treatments face notable limitations in clinical practice: colchicine has a narrow therapeutic window, and glucocorticoids are unsuitable for AGA patients with comorbid diabetes (5, 19). Consequently, developing safe and effective anti-inflammatory drugs remains a critical clinical priority. QZG, a traditional Chinese medicine formula composed of nine herbs, has demonstrated clinical efficacy in alleviating ankle swelling, redness, and pain in patients with AGA (12). Although multiple active components of QZG alleviate inflammation in acute pancreatitis and colitis through immunomodulation (20, 21), whether QZG treats AGA by regulating immune cell function remains unclear. Here, we show that QZG alleviates local joint inflammation in an MSU-induced AGA mouse model. Mechanistically, QZG acts by attenuating HIF-1α-dependent glycolysis, thereby inhibiting the pathogenic polarization of macrophages toward an M1 phenotype, which may contribute to its therapeutic effects.

AGA is triggered by precipitation and deposition of MSU crystals within joints, a result of hyperuricemia (22). Clinically, severe pain, erythema and swelling emerge abruptly hours after MSU crystal deposition, peaking at 24 h (23). Without treatment, the flare typically resolves spontaneously over 7–10 days (24). The intra-articular injection of MSU crystals into the mouse ankle is a well-established model for AGA, as it effectively recapitulates the clinical progression and core pathology observed in humans (25). Given that the first 24 h post-injection represents a critical window for assessing acute inflammation in this model, we pretreated mice with QZG for three days. Model induction was conducted following the fourth dose to evaluate its therapeutic efficacy. Here, our results show that QZG treatment significantly ameliorated ankle swelling, lowered the levels of pro-inflammatory cytokines (IL-1β, TNF-α, and IL-6), and markedly inhibited local immune cell infiltration at 24 h post-modeling.

Network pharmacology has recently emerged as a powerful approach for uncovering the molecular mechanisms underlying traditional Chinese medicine (26, 27). Based on the established anti-AGA efficacy of QZG, we further employed a network pharmacology strategy to explore its potential mechanisms. Firstly, UPLC-Q-TOF-MS was utilized to identify the active components in QZG, and potential targets of these components were predicted using the PubChem and SwissTargetPrediction databases. Meanwhile, differential expression analysis was performed on AGA-related microarray datasets (GSE190138) from the GEO database to identify disease-associated DEGs. By intersecting the predicted therapeutic targets of QZG with AGA-related DEGs, we obtained a set of overlapping genes. Subsequent pathway enrichment analysis of these overlapping genes revealed macrophage polarization as an underlying mechanism mediating QZG’s therapeutic effects against AGA.

Macrophages play a pivotal role in the early inflammatory response to AGA (28). Following intra-articular MSU crystal deposition, macrophages rapidly polarize toward the pro-inflammatory M1 phenotype within the first 24 h (29). These activated cells release a cascade of pro-inflammatory mediators that recruit neutrophils and amplify local inflammation, directly contributing to the clinical hallmarks of redness, swelling, and pain (30, 31). However, the M1-dominant state is not sustained. With inflammatory progression, the rising levels of anti-inflammatory mediators like IL-10 and TGF-β in response to the inflammation actively promote macrophage polarization toward the M2 phenotype (32, 33). After approximately 48 h, the M2 phenotype begins to predominate, initiating the resolution phase of inflammation and facilitating tissue repair (34). Consequently, inhibiting macrophage polarization toward the M1 phenotype or promoting their polarization toward the M2 phenotype is a key therapeutic strategy for AGA (35, 36). In this study, we quantified the proportions of M1 and M2 macrophages in the ankle joints of AGA mice at 24 h post-MSU injection. The results revealed that QZG significantly inhibited M1 macrophage polarization without affecting M2 polarization. To further explore the direct effect of QZG, we established an *in vitro* M1 polarization model using MSU-challenged RAW264.7 cells. Consistent with the *in vivo* findings, QZG treatment markedly suppressed MSU-induced M1 polarization in this cellular model. We speculate that this phenomenon may be attributed to the fact that our current study only evaluated the anti-AGA effects of QZG within 24 h after MSU injection, which corresponds to the acute flare phase of AGA. During this acute stage, M1 macrophages predominate and drive excessive inflammatory progression, and restraining M1 polarization serves as a primary anti-inflammatory strategy for QZG at this time point. By contrast, 48 h after MSU injection, M2 macrophages accumulate progressively. It remains to be clarified whether QZG facilitates M2 polarization and repairs inflammatory lesions in this late phase.

The phenotypic fate of macrophages is intimately linked to their metabolic state, with a reprogramming toward glycolysis being essential for the pro-inflammatory M1 polarization (37). Driven by MSU, macrophages shift their energy supply from oxidative phosphorylation to glycolysis, upregulating a coordinated enzyme cascade to meet the high metabolic demands of inflammation (38, 39). This involves enhanced glucose uptake via GLUT1 (40) and accelerated catalysis by HK2, PFKM, PKM2, PFKFB3 and LDHA (10). This metabolic reprogramming enables rapid energy production and accumulates metabolites such as succinate, thereby promoting M1 macrophage proliferation and pro-inflammatory cytokine secretion (41). Exciting evidence reveals that suppression of macrophage glycolysis restrains M1 polarization. As a representative glycolytic inhibitor, 2-deoxyglucose (2-DG) decreases ECAR and abrogates M1 polarization in LPS-stimulated macrophages (42). In our study, QZG exhibited similar anti-glycolytic effects. Seahorse metabolic assays demonstrated that QZG markedly elevated OCR while decreasing ECAR. Consistently, QZG downregulated the expression of glycolysis-associated genes and proteins both *in vitro and in vivo*, suggesting that QZG functions as a potent suppressor of glycolysis in macrophages.

**Figure 10:**
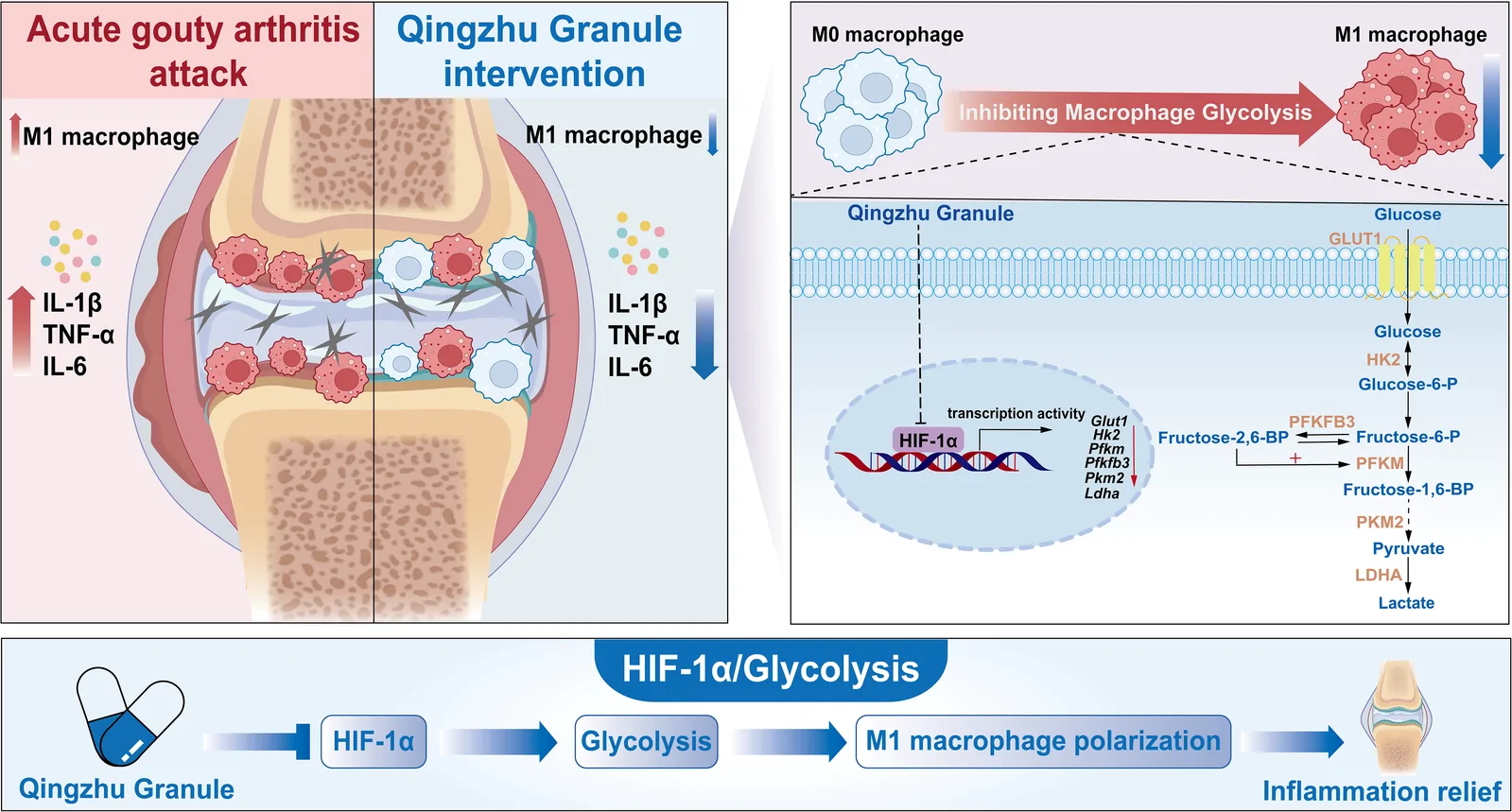
A schematic representation of the identified mechanism of action of QZG treating AGA.

Bioinformatics approaches, including protein-protein interaction (PPI) network analysis and random forest algorithms, are instrumental in identifying critical disease-associated genes and pathways (43). We implemented these analytical approaches on QZG-AGA DEGs and validated the findings using an independent AGA transcriptomic dataset, which identified HIF-1α as a promising target of QZG. HIF-1α is a transcription factor stabilized under hypoxic or pro-inflammatory signals (44). Existing evidence indicates that HIF-1α modulates inflammatory responses via governing macrophage glycolysis. Specifically, HIF-1α overexpression augments macrophage glycolysis and promotes the secretion of pro-inflammatory cytokines (45). Conversely, blockade of HIF-1α reduces the expression of glycolytic enzymes, suppresses M1 macrophage polarization, and ameliorates inflammatory injury (46). In the present study, QZG treatment suppressed HIF-1α expression in AGA mice ankle joints and MSU-stimulated macrophages. Further *in vitro* rescue experiments verified that QZG suppresses MSU-triggered glycolysis and M1 polarization in macrophages by downregulating HIF-1α expression. Therefore, our results delineate a coherent pathway wherein QZG alleviates AGA by attenuating HIF-1α, which downstream curtails glycolytic metabolism and the resultant M1 macrophage-driven inflammation (**Figure 10**).

As expected, QZG-mediated inhibition of the HIF-1α/glycolysis axis exclusively affects M1 macrophage polarization without altering the M2 macrophage phenotype. This discrepancy is attributed to distinct metabolic patterns between M1 and M2 macrophages. M1 macrophages preferentially rely on glycolysis for energy production, whereas M2 macrophages mainly depend on mitochondrial oxidative phosphorylation (10). Under inflammatory conditions, elevated HIF-1α promotes glycolytic enzymatic reactions and energy generation, thereby driving M1 macrophage polarization (45, 46). However, whether the HIF-1α/glycolysis axis participates in the regulation of M2 macrophage polarization remains unclear.

The present study confirmed that QZG markedly downregulates HIF‑1α expression, whereas its regulatory effects on HIF‑1α stability and transcriptional activity remain unclear. To date, no studies have verified whether MSU stimulation modulates HIF‑1α stability and transcriptional activity. Evidence from other inflammatory models indicates that: at the level of protein stability, activation of the PI3K/AKT/mTOR pathway facilitates HIF‑1α translation and improves its stability, thereby inducing M1 macrophage polarization and driving inflammatory progression (47, 48); in terms of transcriptional activity, upregulated Atg5 enhances HIF‑1α transcriptional activity in necrotizing enteritis models, which promotes glycolysis in immune cells and aggravates inflammatory responses (49); at the gene transcription level, LPS and reactive oxygen species mediate the activation of NF-κB, which binds to the *Hif1a* promoter to promote *Hif1a* mRNA transcription (50). Considering our experimental finding that QZG significantly reduces *Hif1a* mRNA expression, subsequent experiments will focus on detecting the activation level of the upstream key protein NF-κB. Meanwhile, we will further investigate the effects of QZG on HIF‑1α stability and transcriptional activity, as well as its upstream regulatory pathways, to elucidate its molecular mechanisms underlying AGA treatment.

## 5 Conclusion

Our study demonstrates that QZG alleviates inflammation by regulating macrophage metabolism within the local microenvironment of AGA, establishing a theoretical basis for the treatment of AGA with QZG. We discovered that QZG inhibits the glycolytic metabolic reprogramming of macrophages by suppressing HIF-1α, thereby preventing their polarization toward the pro-inflammatory M1 phenotype.

## Data Availability

RNA‑seq datasets GSE160170 and GSE190138 were retrieved from the Gene Expression Omnibus (GEO) database (https://www.ncbi.nlm.nih.gov/geo/query/acc.cgi?acc=GSE160170; https://www.ncbi.nlm.nih.gov/geo/query/acc.cgi?acc=GSE190138) Frontiers. Protein‑protein interaction (PPI) analysis was performed using the STRING database (Available at: https://string‑db.org/) Frontiers. Random‑forest algorithm and Venn diagram visualization were implemented via the Hiplot web‑tool (https://hiplot.com.cn/home/index.html). Upstream transcription factors of glycolysis‑related genes were predicted using the ChEA3 database (https://maayanlab.cloud/chea3/) Frontiers. All datasets generated in the present study are available within this article.
