# Correction: Qingzhu granule mitigates acute gouty arthritis through HIF-1a-dependent glycolytic metabolic reprogramming of M1 macrophages

**DOI:** 10.3389/fimmu.2026.1982099

**Published:** 2026-09-11

**Authors:** Yulin Qi, Wenwen Li, Qing Wang, Shuiping Zhou, Yuhong Li, Lin Li

**Affiliations:** 1 Key Laboratory of Traditional Chinese Medical Pharmacology, Tianjin University of Traditional Chinese Medicine, Tianjin, China; 2 State Key Laboratory of Chinese Medicine Modernization, Tasly Pharmaceutical Group Co. Ltd, Tianjin, China; 3 Tianjin Key Laboratory of Component-Based Chinese Medicine, Tasly Pharmaceutical Group Co. Ltd, Tianjin, China; 4 Ministry of Education Key Laboratory of Pharmacology of Traditional Chinese Medical Formulae, Tianjin University of Traditional Chinese Medicine, Tianjin, China; 5 State Key Laboratory of Chinese Medicine Modernization, Tianjin University of Traditional Chinese Medicine, Tianjin, China

**Keywords:** acute gouty arthritis (AGA), glycolysis, HIF-1α, M1 macrophage polarization, Qingzhu granule (QZG)

Authors “Yulin Qi, Wenwen Li and Qing Wang” were erroneously omitted as equal contributing author.

The original version of this article has been updated.

