# Supplementary material for: Qingzhu granule mitigates acute gouty arthritis through HIF-1α-dependent glycolytic metabolic reprogramming of M1 macrophages
Source: Front Immunol. 2026 Aug 26;17:1817105. doi: 10.3389/fimmu.2026.1817105 (PMC13553399; doi:10.3389/fimmu.2026.1817105)
Supplement: Supplementary file 1 [file DataSheet1.pdf]

frontiers

The International Society for the Advancement of Cytometry (ISAC) has highlighted the importance of including comprehensive methodological information to ensure data reproducibility and reliability. In line with this, Frontiers in Immunology now requires authors to submit a checklist for manuscripts that involve flow or mass cytometry. This checklist helps standardize the reporting process, improving the quality and transparency of published data. By doing so, we support scientific progress, making it easier for other researchers to replicate and validate experiments.

This form should be submitted with any manuscripts using flow or mass cytometry.

Sample/specimen/material description

☐ Total blood

☐ PBMCs

☑ Organ digests

Other____

Did the samples suffer any treatment before or after incubation with the antibodies?

☐ Drug _____________________________________

☑ Cell permeabilization ______________________

☐ Dye _______________________________________

☐ Propidium iodine

☐ Not applicable

Other ____

Instrument and antibodies

Name of the Cytometer Attune NxT Flow Cytometer

Antibodies and targets	Fluorochrome/ Metal	Catalog number/Company
e.g. anti-CD4	FITC	Cat. XXX/ XXX ltd.
anti-F480	PE	111603/Biolegend

anti-CD86	APC	159216/Biolegend
anti-CD206	FITC	141703/Biolegend

Data analyses

1. Name of the software FlowJo
2. Reference gating strategy in the manuscript or supplementary material

Gating strategy in (eg Figure X) supplementary material
