# Supplementary material for: Qingzhu granule mitigates acute gouty arthritis through HIF-1α-dependent glycolytic metabolic reprogramming of M1 macrophages
Source: Front Immunol. 2026 Aug 26;17:1817105. doi: 10.3389/fimmu.2026.1817105 (PMC13553399; doi:10.3389/fimmu.2026.1817105)

CD206

CON

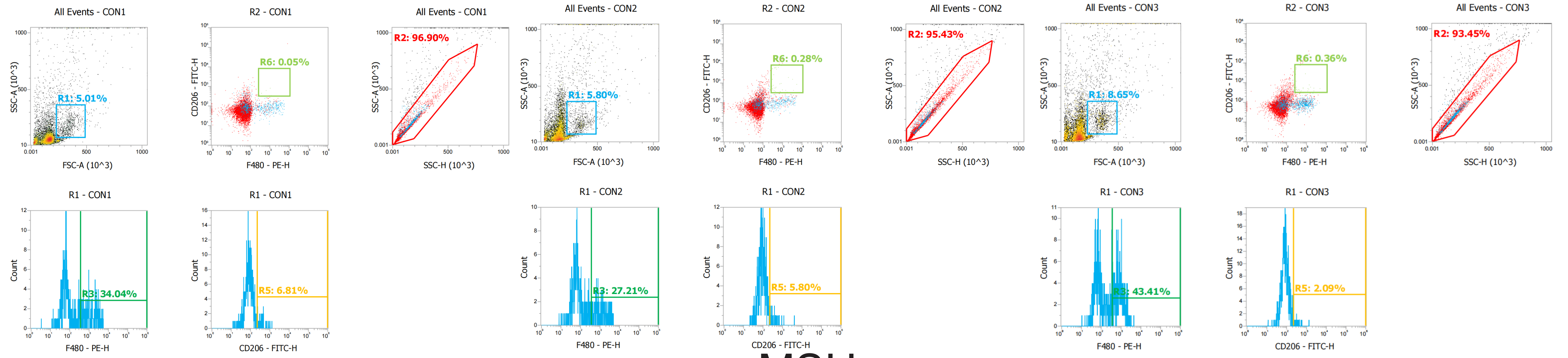

MSU

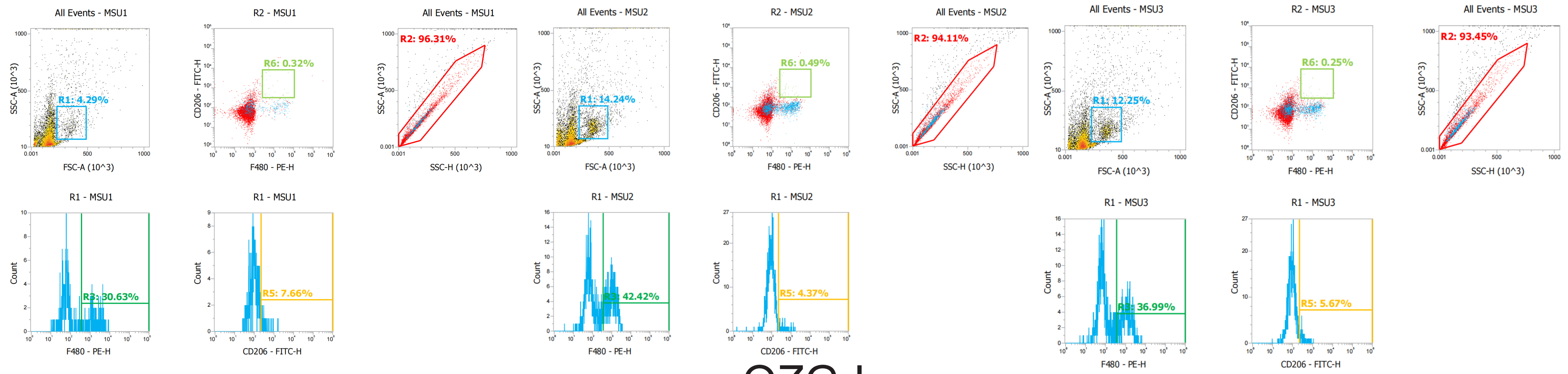

QZG-L

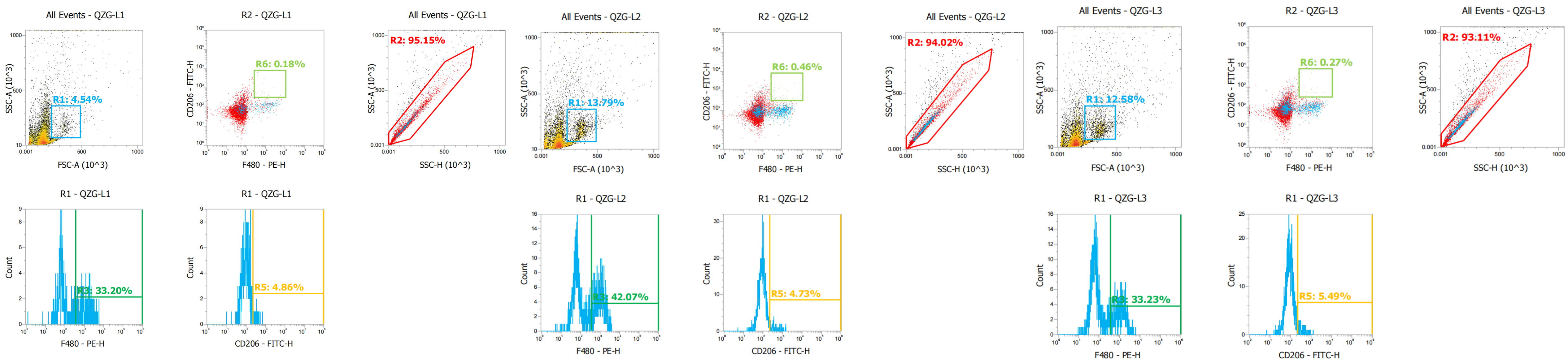

QZG-H

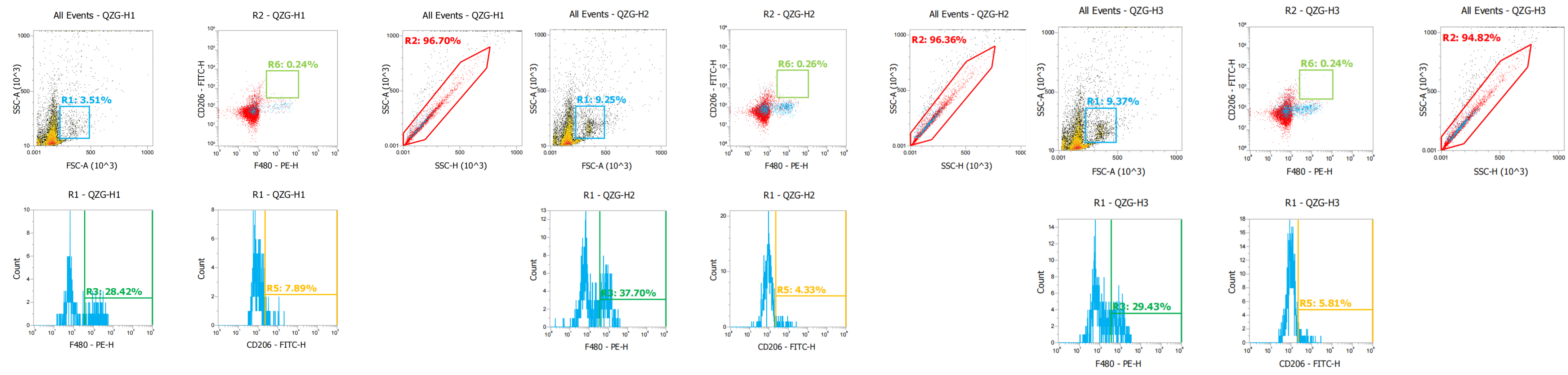

Supplement: Supplementary file 2 [file DataSheet10.pdf]
