# Supplementary material for: Qingzhu granule mitigates acute gouty arthritis through HIF-1α-dependent glycolytic metabolic reprogramming of M1 macrophages
Source: Front Immunol. 2026 Aug 26;17:1817105. doi: 10.3389/fimmu.2026.1817105 (PMC13553399; doi:10.3389/fimmu.2026.1817105)
Supplement: Supplementary file 4 [file DataSheet2.pdf]

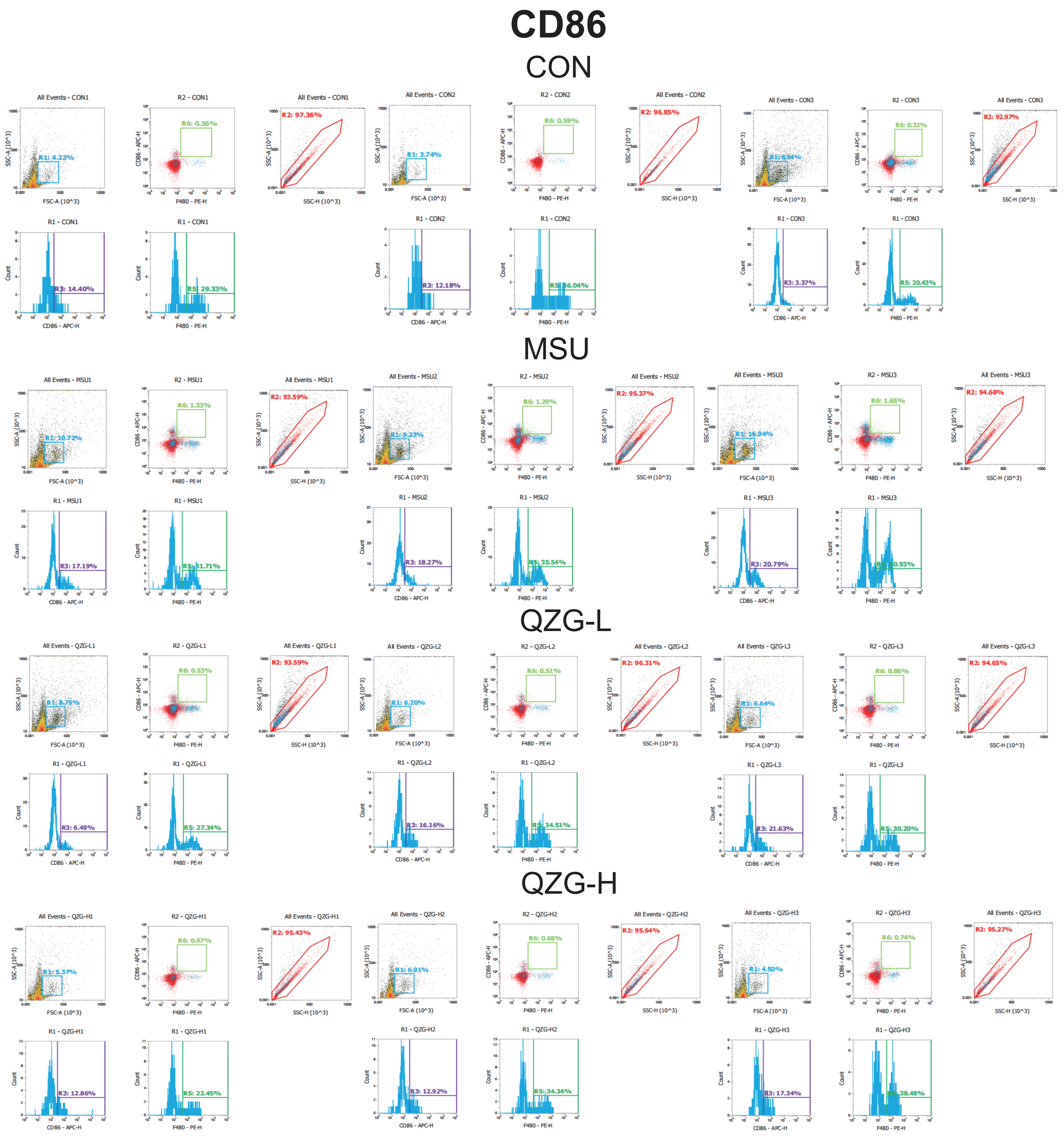

CD86
CON
All Events - CON1
R1: 4.23%
R2 - CON1
R6: 0.36%
All Events - CON1
R2: 97.36%
R1 - CON1
R3: 14.40%
R1 - CON1
R5: 29.33%
All Events - CON2
R1: 3.74%
R2 - CON2
R6: 0.59%
All Events - CON2
R2: 96.85%
R1 - CON2
R3: 12.18%
R1 - CON2
All Events - CON3
R2 - CON3
R6: 0.32%
All Events - CON3
R2: 92.97%
R1 - CON3
R3: 3.37%
R1 - CON3
R5: 20.43%
MSU
All Events - MSU1
R1: 10.72%
R2 - MSU1
R6: 1.33%
All Events - MSU1
R2: 93.59%
R1 - MSU1
R3: 17.19%
R1 - MSU1
All Events - MSU2
R1: 9.23%
R2 - MSU2
R6: 1.29%
All Events - MSU2
R2: 95.37%
R1 - MSU2
R3: 18.27%
R1 - MSU2
R5: 35.54%
All Events - MSU3
R1: 16.94%
R2 - MSU3
R6: 1.65%
All Events - MSU3
R2: 94.68%
R1 - MSU3
R3: 20.79%
R1 - MSU3
QZG-L
All Events - QZG-L1
R2 - QZG-L1
R6: 0.53%
All Events - QZG-L1
R2: 93.59%
R1 - QZG-L1
R3: 6.49%
R1 - QZG-L1
R5: 27.34%
All Events - QZG-L2
R1: 6.20%
R2 - QZG-L2
R6: 0.51%
All Events - QZG-L2
R2: 96.31%
R1 - QZG-L2
R3: 16.16%
R1 - QZG-L2
R5: 34.51%
All Events - QZG-L3
R1: 6.64%
R2 - QZG-L3
R6: 0.86%
All Events - QZG-L3
R2: 94.65%
R1 - QZG-L3
R3: 21.63%
R1 - QZG-L3
R5: 30.20%
QZG-H
All Events - QZG-H1
R1: 5.37%
R2 - QZG-H1
R6: 0.67%
All Events - QZG-H1
R2: 95.43%
R1 - QZG-H1
R3: 12.86%
R1 - QZG-H1
R5: 22.45%
All Events - QZG-H2
R1: 6.91%
R2 - QZG-H2
R6: 0.68%
All Events - QZG-H2
R2: 95.64%
R1 - QZG-H2
R3: 12.92%
R1 - QZG-H2
R5: 34.34%
All Events - QZG-H3
R1: 4.50%
R2 - QZG-H3
R6: 0.74%
All Events - QZG-H3
R2: 95.27%
R1 - QZG-H3
R3: 17.34%
R1 - QZG-H3
R5: 38.48%
SSC-A (10^3)
FSC-A (10^3)
SSC-H (10^3)
CD86 - APC-H
F480 - PE-H
Count
