# Supplementary material for: Qingzhu granule mitigates acute gouty arthritis through HIF-1α-dependent glycolytic metabolic reprogramming of M1 macrophages
Source: Front Immunol. 2026 Aug 26;17:1817105. doi: 10.3389/fimmu.2026.1817105 (PMC13553399; doi:10.3389/fimmu.2026.1817105)
Supplement: Supplementary file 5 [file Table1.docx]

**Supplementary Tables**

**Table S1**

| Number | Name | Molecular Formula | m/z | *t_R_*/min |
| --- | --- | --- | --- | --- |
| 1 | higenamine glucoside | C_22_H_27_NO_8_ | 434.1812[M+H]^+^ | 4.92 |
| 2 | laudanosoline-1-  *O*-xylopyranose | C_22_H_27_NO_8_ | 434.1804[M+H]^+^ | 5.14 |
| 3 | acutumine | C_18_H_22_ClNO_6_ | 406.1034[M+Na] ^+^ | 8.20 |
| 4 | 3’-hydroxy-*N*-  methylcoclaurine  -1-*O*-glicoside | C_24_H_31_NO_9_ | 478.2079[M+H]^+^ | 8.70 |
| 5 | sinomenine | C_19_H_23_NO_4_ | 330.1716[M+H]^+^ | 9.45 |
| 6 | ferulic acid | C_10_H_10_O_4_ | 193.0500[M-H]^-^ | 10.45 |
| 7 | fabiatrin | C_21_H_26_O_13_ | 485.1273[M-H]^-^ | 11.73 |
| 8 | phellodendrine | C_20_H_24_NO_4_ | 342.1716[M]^+^ | 12.54 |
| 9 | tembetarine | C_20_H_26_NO_4_ | 344.1862[M]^+^ | 13.53 |
| 10 | magnolflorine | C_20_H_24_NO_4_ | 342.1708[M]^+^ | 14.06 |
| 11 | 3-O-Feruloylquinic or its isomers | C_17_H_20_O_9_ | 367.1033[M-H]^-^ | 14.67 |
| 12 | 3-O-Feruloylquinic or its isomers | C_17_H_20_O_9_ | 367.1035[M-H]^-^ | 14.89 |
| 13 | magnolflorine isomers | C_20_H_24_NO_4_ | 342.1707[M]^+^ | 15.89 |
| 14 | oblongine | C_19_H_23_NO_3_ | 314.1759[M+H]^+^ | 16.09 |
| 15 | unknown | C_19_H_20_O_4_ | 312.1566[M]^+^ | 16.41 |
| 16 | syringaresinol-di-O-glucoside | C_36_H_46_O_8_ | 765.2568[M+Na]^+^ | 17.28 |
| 17 | rotundine | C_21_H_25_NO_4_ | 356.18675[M+H]^+^ | 17.99 |
| 18 | polydatin | C_20_H_22_O_8_ | 413.1215[M+Na]^+^ | 18.30 |
| 19 | neoeriocitrin | C_27_H_32_O_15_ | 619.1627[M+Na]^+^ | 19.49 |
| 20 | cepharanthine | C_19_H_18_NO_4_ | 324.1244[M]^+^ | 20.38 |
| 21 | naringin isomers | C_27_H_32_O_14_ | 603.1684[M+Na]^+^ | 21.37 |
| 22 | naringin | C_27_H_32_O_14_ | 603.1686[M+Na]^+^ | 22.48 |
| 23 | hesperidin | C_28_H_34_O_15_ | 633.1799[M+Na]^+^ | 23.28 |
| 24 | neohesperidin | C_28_H_34_O_15_ | 633.1793[M+Na]^+^ | 24.33 |
| 25 | modin1-glucoside | C_21_H_20_O_10_ | 431.0953[M-H]^-^ | 25.17 |
| 26 | salvianolic acid B | C_36_H_30_O_16_ | 717.1433[M-H]^-^ | 27.25 |
| 27 | berberine | C_20_H_18_NO_4_ | 336.1243[M]^+^ | 29.02 |
| 28 | modin1-glucoside isomers | C_21_H_20_O_10_ | 431.0978[M-H]^-^ | 30.75 |
| 29 | dioscorosides B2 | C_52_H_86_O_24_ | 1093.5444[M-H]^-^ | 31.58 |
| 30 | Protogracillin | C_51_H_84_O_23_ | 1063.5343[M-H]^-^ | 31.73 |

**Table S1** Identification Results of Main Components of QZG Samples

**Table S2**

**Table S2** Primer sequences of genes

| Genes | Forward primer; reverse primer (5’-3’) |
| --- | --- |
| *Hif-1α* | Forward-cagccagcaagtccttctga  Reverse-cactgggactgttaggctgg |
| *Glut1* | Forward-cactgtggtgtcgctgtttg  Reverse-aaagatggccacgatgctca |
| *Hk2* | Forward-caccctacagcagctgtgaa  Reverse-tctccatctccaccctctgg |
| *Pfkm* | Forward-ttggagctgatggaaggcag  Reverse-tgcagactgcttgattcggt |
| *Pkm2* | Forward-tcaccctggacaacgcttac  Reverse-agtcagcgcctttctccttc |
| *Ldha* | Forward-agtaagtcctcaggcggcta  Reverse-tgagggttgccatcttggac |
| *Pfkfb3* | Forward-tgtccagcagaggcaagaag  Reverse-tctcgttgagtgccttccac |
